# Mechanisms of cellular senescence-induced vascular aging: evidence of senotherapeutic strategies

**DOI:** 10.20517/jca.2024.31

**Published:** 2025-03-19

**Authors:** Sophia A. Mahoney, Samuel I. Bloom, Douglas R. Seals, Anthony J. Donato, Matthew J. Rossman, Zachary S. Clayton

**Affiliations:** 1 Department of Integrative Physiology, University of Colorado Boulder, Boulder, CO 80309, USA.; 2 Salk Institute for Biological Studies, La Jolla, CA 90237, USA.; 3 Department of Internal Medicine-Geriatrics, University of Utah, Salt Lake City, UT 84113, USA.; 4 Department of Medicine-Geriatrics, University of Colorado Anschutz Medical Campus, Aurora, CO 80045, USA.

**Keywords:** Vascular dysfunction, cellular senescence, senotherapeutics, vascular aging

## Abstract

Cardiovascular diseases (CVD) remain the leading cause of death worldwide, with advancing age being the primary, nonmodifiable risk factor. Vascular dysfunction, namely arterial stiffening and endothelial dysfunction, is the key antecedent to the development of clinical CVD with aging. Fundamental aging macro-mechanistic processes that drive vascular aging include excess oxidative stress, chronic inflammation, and declines in the vasodilatory molecule nitric oxide. An important hallmark of aging that contributes to the vascular aging processes is cellular senescence - a stress response characterized by cell cycle arrest and accompanied by the production and secretion of proinflammatory molecules (i.e., the senescence-associated secretory phenotype [SASP]). Excess senescent cells and the SASP have deleterious effects on vascular function and in states of CVD, making it a putative therapeutic target for improving vascular function and preventing or reversing CVD. This review will focus on the role of cellular senescence in age-related vascular dysfunction and CVD. We will examine established and emerging mechanisms underlying cellular senescence-induced vascular dysfunction. We will then discuss groups with impaired vascular function and high cellular senescence burden and examine strategies to reduce or remove excess senescent cells and the SASP in the groups who are likely to benefit most from these therapies. Finally, we will highlight the systemic effects of vascular senescent cell suppression on other tissues and organs, given the integrative role of the vasculature in physiology. Together, this review will underscore the imperative role of cellular senescence in vascular dysfunction and the need for a deeper understanding of the translational use of cellular senescence and SASP targeting therapies in groups with high senescent cell burden.

## Graphical Abstract

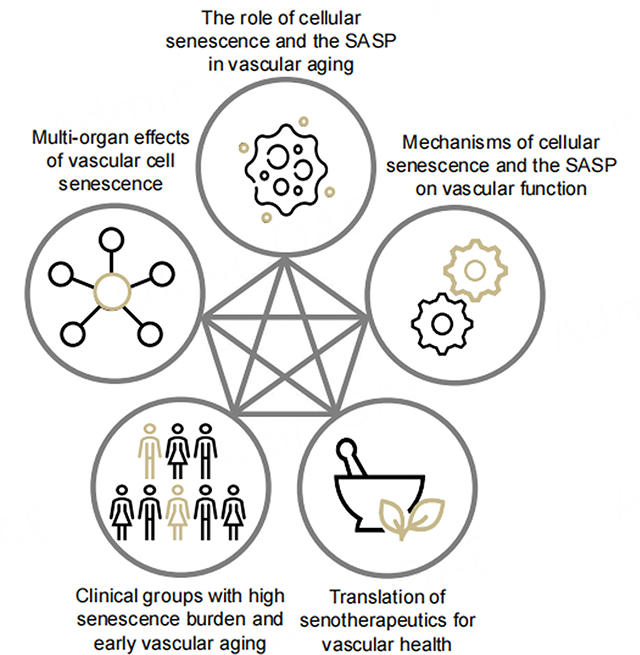

## INTRODUCTION

Cardiovascular diseases (CVD) are the leading cause of death worldwide, and despite major advances in CV medicine, advancing age remains the major CVD risk factor^[1–3]^. Demographic projections predict continuous increases in the number and percentage of older adults (i.e., people over 65 years of age) over the next several decades^[4]^. The combination of age as a risk factor for CVD and the projected increase in the number of older adults will lead to a higher incidence of CV events and a greater prevalence of CVD. As such, it is crucial to identify novel mechanisms of CVD and therapeutics to prevent and treat CVD.

Advanced age results in vascular dysfunction characterized by the stiffening of the large elastic arteries (i.e., aorta) and impaired vascular endothelial function, both of which are key antecedents of CVD^[5]^. Age-related vascular dysfunction is a consequence of several fundamental macro-mechanistic processes, including chronic inflammation, excess oxidative stress, and declines in the bioavailability of the vasodilatory molecule nitric oxide (NO)^[6–8]^. While the etiology of these fundamental macro-mechanistic processes has not been fully elucidated, the processes have been well characterized, and modulating these pathways improves vascular function in older mammals^[9,10]^. Cellular senescence, a hallmark of aging, has emerged as a plausible contributor to inflammation, oxidative stress, and suppressed NO^[8]^. Cellular senescence is a stress response characterized by stable cell cycle arrest and the production of a proinflammatory milieu referred to as the senescence-associated secretory phenotype (SASP)^[11]^. Advancing age increases the abundance of senescent cells in the vasculature^[12]^, which contributes to age-related vascular dysfunction^[13]^ and several CVD^[14]^. Therefore, targeting cellular senescence and the SASP using senotherapeutics - therapies that suppress excess senescent cells and/or the SASP - is an appealing strategy to treat and prevent vascular dysfunction and mitigate CVD risk^[8]^.

In this review, we will discuss: (1) a summary of the role of cellular senescence and the SASP in vascular dysfunction; (2) established and putative mechanisms through which excess senescent cells and the SASP drive vascular dysfunction; (3) clinical groups with high senescence burden that may benefit most from senotherapeutics; and (4) translational challenges in senotherapeutic dosing and clinical implementation.

### AGE-RELATED VASCULAR DYSFUNCTION AND MECHANISMS OF VASCULAR AGING

Large elastic artery stiffening and vascular endothelial dysfunction are two independent predictors of future CV events and mortality^[15,16]^. Stiffening of the large elastic arteries (i.e., the aorta) occurs mostly through changes in the mechanical properties of the vessel wall^[17]^. During aging, vascular smooth muscle cells (VSMCs) undergo phenotypic switching from synthetic to contractile states, promoting unfavorable vascular remodeling such as changes in the stress/strain components of the arteries^[18]^. Aortic stiffness is assessed non-invasively in mice and humans by the reference standard *in vivo* measure of pulse wave velocity (PWV)^[19]^. This assessment quantifies the stiffness of the aorta by measuring the time elapsed for the pressure wave produced by the contraction of the heart during systole to propagate bidirectionally to the two pulse pressure sites^[19]^. Importantly, PWV increases with advancing age and increases pressure pulsatility, thereby affecting systolic blood pressure in a feed-forward cycle^[17]^. As such, increased arterial stiffness contributes to hypertension and is independently predictive of future CVD^[16]^.

Endothelial dysfunction is another primary manifestation of vascular aging characterized, in part, by impaired endothelium-dependent dilation (EDD)^[20]^. Endothelial cells make up a monolayer that lines the inner-most wall of blood vessels^[21]^. In response to mechanical shear stress induced by antegrade blood flow or exposure to pharmacological agents (i.e., acetylcholine), endothelial cells produce NO, which diffuses into the VSMC to cause vasodilation and regulate vascular tone^[22]^. In humans, EDD is commonly measured using the non-invasive reference-standard approach of brachial artery flow-mediated dilation (FMD), which is primarily dependent on the endothelial response to produce NO, and is a widely used measure to characterize the health of the macrovascular endothelium^[15]^. Another key characteristic of age-related endothelial dysfunction includes dysregulation of delivery of essential nutrients and oxygen to peripheral tissues^[21]^. Given that the endothelium lines the circulation, endothelial cells serve as an important barrier and regulator of biological factors that cross into peripheral tissues^[21]^.

Fundamental macro-mechanistic processes underlying arterial stiffening and endothelial dysfunction include excess oxidative stress, chronic, low-grade inflammation, and reduced NO bioavailability^[23]^. With aging, oxidative stress results from a combination of excess reactive oxygen species (ROS) production and decreased antioxidant defenses due primarily to reductions in antioxidant enzyme activity/abundance^[9,24]^. Excess ROS readily reacts with NO to form peroxynitrite and lower NO bioavailability^[24]^. Peroxynitrite may also oxidize tetrahydrobiopterin (BH4), an essential cofactor for NO production by endothelial NO synthase (eNOS)^[25]^. Importantly, this results in eNOS uncoupling, whereby eNOS generates ROS instead of NO^[25]^. Together, these processes promote ROS bioactivity and reduce NO bioavailability to impair endothelial function^[20]^. Excess ROS may also activate proinflammatory and prooxidant signaling pathways^[26]^. This intrinsic inflammatory activation in the microenvironment or in the circulation can activate other ROS-producing systems, creating a feed-forward cycle of inflammation and oxidative stress that exacerbates vascular dysfunction. States of high oxidative stress and inflammation contribute to arterial stiffening by promoting adverse structural remodeling of the vascular walls^[8]^. Notably, four unfavorable structural changes occur in the vascular wall with aging: (1) there is a degradation and fragmentation of elastin; (2) there is a compensatory deposition of collagen to replace the fractured elastin; (3) the stimulation of advanced glycation end products (AGEs) promotes crosslinking of these structural components; and (4) AGE-induced calcium (Ca^2+^) deposition leading to arterial calcification^[8,27]^. There are also functional contributions to arterial stiffening driven by reduced vasodilatory factors (i.e., NO and hyperpolarizing factors) and increased vasoconstrictive factors (i.e., angiotensin II) that confer VSMC stiffness^[8,28]^. Together, these processes reduce elasticity and contribute to the stiffness of the arteries. Although these major macromechanistic events are well-accepted as the underlying mechanisms of vascular aging^[23]^, the integrative cellular/molecular processes regulating excessive oxidative stress, chronic inflammation, and limited NO bioavailability with aging are incompletely understood.

### THE ROLE OF CELLULAR SENESCENCE IN VASCULAR DYSFUNCTION

**Figure 1.**
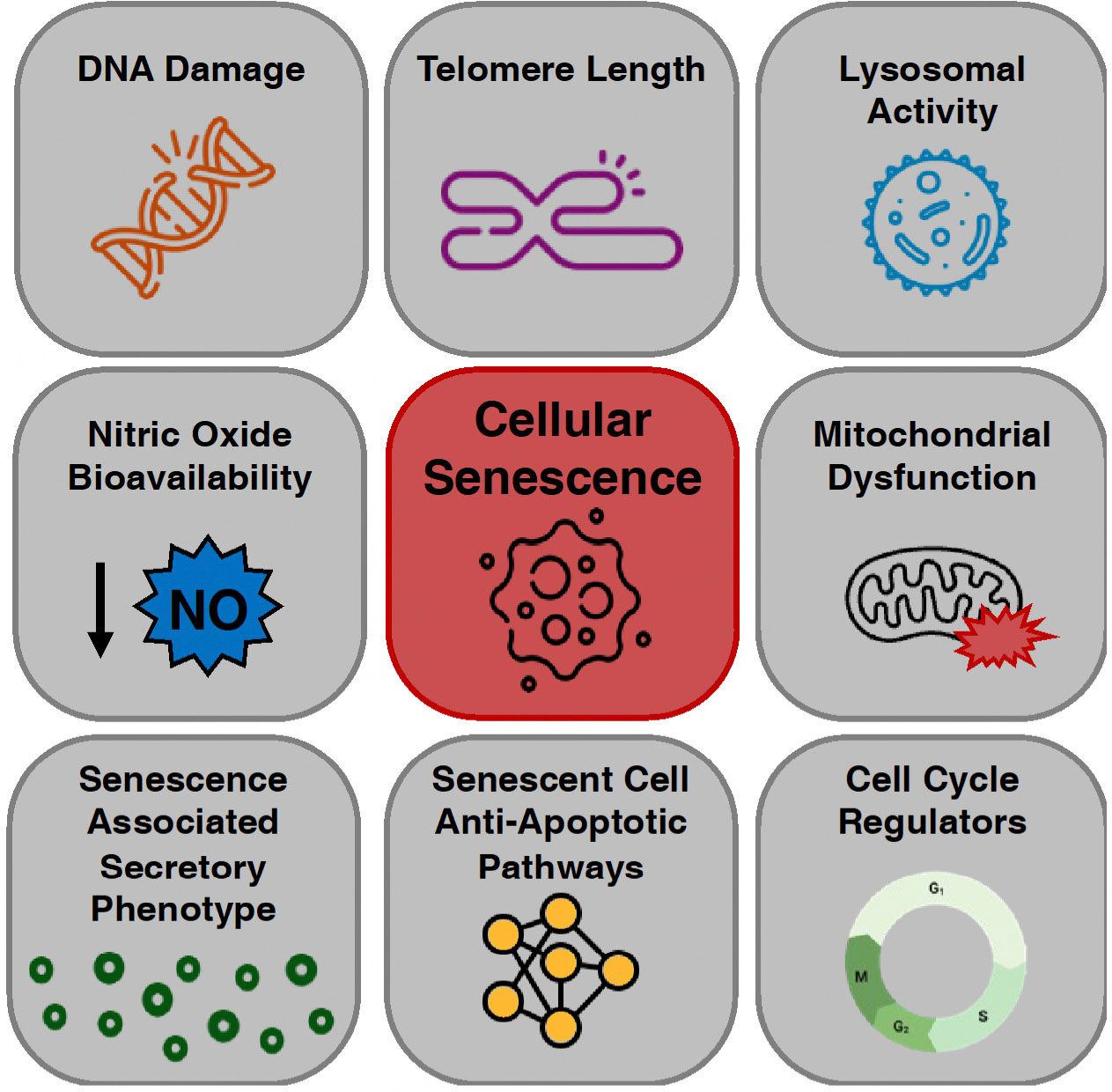
Characteristics of a vascular senescent cell. Vascular cells including endothelial cells and vascular smooth muscle cells undergo phenotypic changes upon cellular senescence induction. These senescent cell characteristics are leveraged to identify senescent cells in the vasculature.

Cellular senescence is a complex biological process characterized by the irreversible growth arrest of cells in response to various stimuli, including DNA damage, telomere shortening, oxidative stress, and oncogene activation^[11,29]^. Senescent cells exhibit a distinct phenotype compared to healthy, proliferating cells, including an enlarged and flattened morphology, increased metabolic activity, and an amplified secretion of a wide range of proinflammatory cytokines, chemokines, and growth factors collectively referred to as the senescence-associated secretory phenotype (SASP)^[30,31]^. Senescent cells and the SASP contribute to many physiological processes, including embryonic development, wound healing, and tumor suppression^[29,32]^. However, the accumulation of senescent cells and the SASP has been implicated in various age-related pathologies, including CVD^[33–35]^. Increased cellular senescence is evident in clinical vascular pathologies including atherosclerosis^[36,37]^, pulmonary hypertension^[38]^, and aortic aneurysms^[39]^. In the absence of disease, the vasculature remains the tissue with the greatest senescent cell burden in old mice and in models of premature aging in mice^[12]^, suggesting that the vasculature is particularly vulnerable to cellular senescence. Key vascular cells, such as endothelial cells^[40–42]^ and VSMCs^[43,44]^, become senescent with chronological aging and with other stressors *in vivo*. Importantly, vascular senescent cells are identified by key characteristics and biomarkers including cell cycle regulators (i.e., *cdkn1a* - p21, *Cdkn2a* - p16 and p19), altered lysosomal activity (senescence-associated β-galactosidase [SA-β-Gal]), DNA damage markers (53BP1 and γ-H2AX), telomere length and telomerase activity, senescent cell anti-apoptotic pathways (SCAPs), and SASP profile (IL-6, IL-1β, and tumor necrosis factor [TNF-], among many others)^[31,45]^ [Figure 1]. Together, this evidence suggests that the burden of cellular senescence is greatest in the vasculature compared to other tissues and contributes to impairments in function and the development of CVD. Therefore, targeting senescence in the vasculature may improve function and reduce age-related vascular pathologies. Below, we discuss mechanisms of vascular cell senescence, groups with high senescent cell burden, and therapeutic treatments that target senescent cells to promote healthy vascular aging.

### ESTABLISHED AND EMERGING MECHANISMS UNDERLYING CELLULAR SENESCENCE-INDUCED VASCULAR DYSFUNCTION

#### Established mechanisms of vascular senescence

**Figure 2.**
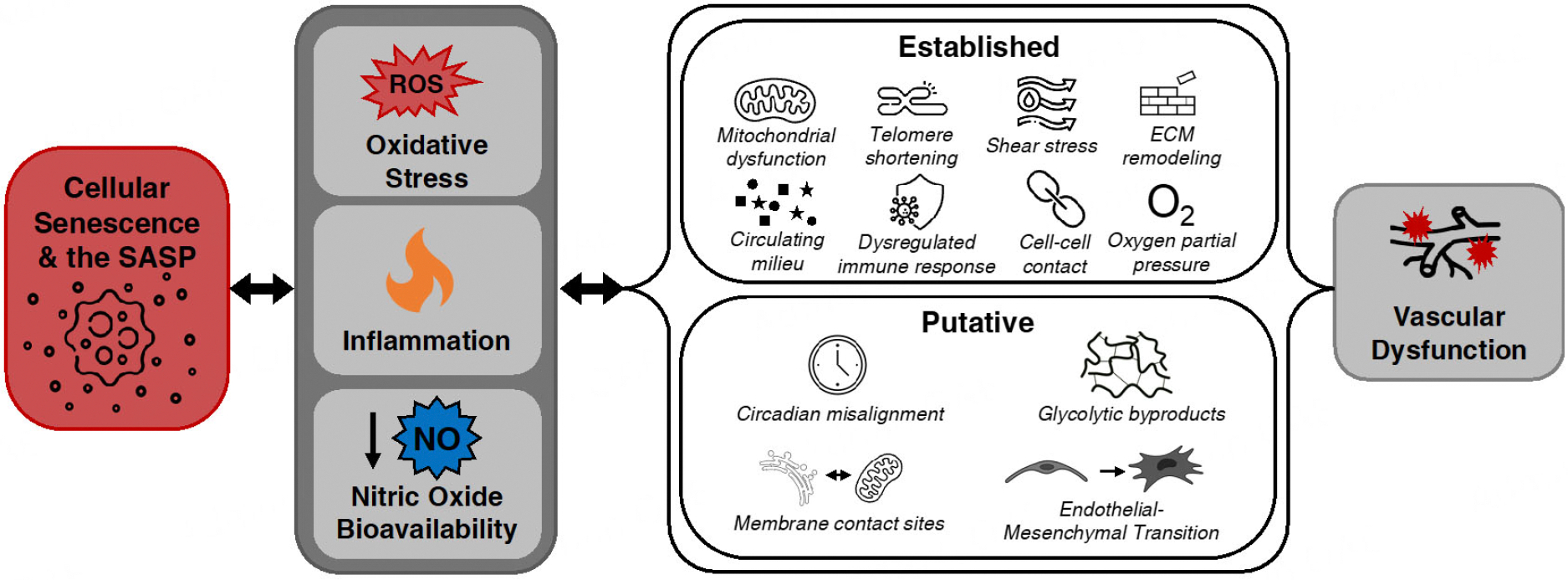
Mechanisms by which cellular senescence contributes to vascular dysfunction. Excess senescent cell and senescence-associated secretory phenotype (SASP) burden drive macro-mechanistic processes including reactive oxygen species (ROS)-related oxidative stress, chronic inflammation, and reduced nitric oxide (NO) bioavailability. In turn, these fundamental aging processes promote other established and putative underlying mechanisms that contribute to vascular dysfunction. ECM: Extracellular matrix; ER: endoplasmic reticulum.

Cellular senescence has recently been causally established as a contributor to age-related vascular dysfunction. Many of the underlying mechanisms of vascular dysfunction and vascular cell senescence overlap, providing unique therapeutic targets that improve vascular function by reducing senescent cell burden. Select underlying processes have been established supporting the mechanisms by which senescent cells contribute to vascular dysfunction and serve as therapeutic targets for senescent cells^[46]^. Here, we discuss studies that investigate the established mechanisms by which senescent cells contribute to vascular dysfunction, and then discuss putative mechanisms that have recently emerged as novel means by which senescent cells elicit their detrimental effects on vascular physiology [Figure 2].

##### Mitochondrial dysfunction

Mitochondrial dysfunction, characterized in part by reduced electron transport system function, impaired mitophagic capacity, and elevated ROS production, is a key feature of vascular aging^[47]^. Cellular senescence promotes mitochondrial dysfunction in both an autocrine and paracrine manner as a result of the proinflammatory SASP^[48,49]^. In senescent cells, mitochondria undergo outer membrane permeabilization, which releases mitochondrial DNA into the cytosol^[50]^. In turn, cytosolic mitochondrial DNA activates the cGAS-STING pathway, a major regulator of the SASP^[50]^. Mitochondrial-derived ROS contribute to vascular dysfunction with aging, as evidenced by a reduction in mitochondrial ROS, reduced vascular mitochondrial prooxidant markers, and a higher abundance of mitochondrial antioxidant defense following systemic senescent cell clearance^[13,51]^. Although cellular senescence and mitochondrial dysfunction are both considered hallmarks of aging, the bidirectional relationship between these processes further highlights the multiplicity of benefits of targeting senescent cells. Given the essential role of the mitochondria in senescent cells and in regulating the SASP, dysfunctional mitochondria may serve as an important therapeutic target to mitigate the detrimental effects of cellular senescence and the SASP on vascular function.

##### Telomere shortening and dysfunction

Telomeres, the DNA sequences and proteins that protect the caps of chromosomes, shorten with every replication cycle and are maintained by the enzyme telomerase^[52]^. Loss of telomere due to excess shortening or insufficient telomerase expression is an established cause of stress-induced senescence^[52]^. Endothelial cells have a low replicative capacity, accept at vascular regions where they are exposed to low and oscillatory shear stress, rendering them susceptible to telomere-shortening induced senescence^[52]^. In addition to shortening, telomere dysfunction may occur independent of length, likely due to oxidative stress to directly mediate vascular cell senescence^[40]^. Specifically, endothelial cell telomere dysfunction-induced senescence impairs endothelial function through increased inflammatory signaling and oxidative stress in young mice^[40]^. Further, telomere length is inversely associated with vascular elasticity and CVD events starting in people in mid-life^[53]^. More studies are warranted to determine the direct implications of telomere function/length with arterial stiffening and to extend these findings to people.

##### Shear stress

Endothelial cells are constantly exposed to shear stress created by blood flow. This unique environmental stimulus has pathophysiological implications, which are regional, as blood flow patterns vary widely across the vascular network. At vascular curvatures, branches, and bifurcations, low and oscillatory shear stress impairs endothelial function and is associated with an inflammatory phenotype. Conversely, high and laminar shear stress tends to be vasoprotective^[54]^. In relation to cellular senescence, regions of the circulation exposed to low and oscillatory shear stress exhibit elevated markers of cellular senescence and stimuli such as DNA damage and telomere dysfunction compared to cells that experience high and laminar shear stresses^[40,55–57]^. Importantly, these regions are also prone to the development of atherosclerosis^[55,57–59]^. Mechanistically, low and oscillatory shear stress induces cellular senescence by increasing cell turnover, which is likely exaggerated in the vasculature compared to other cells in the body^[59,60]^. However, in regions exposed to low and oscillatory shear stress, endothelial cells are pushed to divide more rapidly, and clonal hyperplasia of VSMCs contributes to atherosclerotic plaque development^[61–63]^. In endothelial cells of large arteries, the replicative burden is not mitigated by circulating progenitors as cell regeneration requires mitotic cell division by neighboring cells^[64]^. Thus, the vasculature is comprised of long-lived cells that endure long-term exposure to low, oscillatory blood flow and senescence-inducing circulating factors. At vascular bifurcations, however, replicative stress may promote states of cellular senescence.

##### ECM remodeling

As previously mentioned, arterial stiffening is a key pathophysiological process of vascular aging. Senescent vascular cells contribute to arterial stiffness in various capacities, including VSMC stiffness and unfavorable ECM remodeling. Particularly, senescent VSMCs have a higher secretion of ECM proteins and are stiffer and less organized than the ECM derived from young control VSMCs^[27]^. Pro-stiffening changes in the ECM of VSMC are mediated by altered collagen deposition and reduced contractility in senescent VSMCs^[27]^. Sry-box transcription factor 9 (SOX9), a regulator of chondrocyte differentiation, is a central mediator of ECM stiffness and organization in senescent VSMCs^[27]^. Mitochondrial-derived ROS also contributes to vascular dysfunction with aging, as evidenced by a reduction in mitochondrial ROS, reduced vascular mitochondrial prooxidant markers, and a higher abundance of mitochondrial antioxidant defense following systemic senescent cell clearance^[13,51]^. Importantly, senescent cell clearance results in a reduction in collagen and AGE abundance in the vascular wall of old mice^[13,65]^. As such, the vascular ECM is an important contributor to cellular senescence-induced arterial stiffening and a therapeutic target to prevent or reverse unfavorable vascular remodeling.

##### The circulating milieu

The influence of the circulating milieu (i.e., the collection of bioactive factors in systemic circulation) on physiological function has been studied using a variety of experimental approaches including heterochronic parabiosis (surgical joining of two circulatory systems)^[66]^, heterochronic blood exchange (administration of blood/blood factors of one organism into the circulating milieu of another organism)^[67]^, and *in vitro* or *ex vivo* exposure of cells^[68,69]^ or organ systems^[13,70]^, respectively, to distinct components of the circulation (e.g., blood/plasma/serum). Collectively, these experimental approaches have demonstrated that age-related vascular dysfunction can be transferred via the circulating milieu such that the aged circulating milieu promotes cellular senescence in a variety of cell/tissue types in young animals^[13,67,70]^. Notably, age-related vascular dysfunction is prevented in arteries exposed to the circulating milieu collected from old animals following senescent cell clearance^[71]^. Individual circulating factors, including nutrients, metabolites, and hormones, are also capable of inducing cellular senescence. A large body of evidence from a variety of models suggests that glucose, amino acids, lipids, and insulin are capable of provoking senescent states (reviewed in detail elsewhere)^[72]^. Together, these results demonstrate that the circulating milieu mediates vascular aging, and this may occur, in part, via cellular senescence and changes in the circulating SASP. However, future studies are warranted to determine a mechanistic role of the circulating SASP factors implicated in vascular dysfunction^[73,74]^, which are responsible for increasing cellular senescence in the vasculature and transducing the vascular aging phenotype.

##### Dysregulated immune responses

An additional characteristic that predisposes vascular cells to damage is their interaction with the interstitial microenvironment and neighboring cells. Inflammatory immune cells traffic through the vascular network, and endothelial cells mediate extraversion from the circulation to the extravascular space^[75]^. Endothelial cells can upregulate the expression of adhesion molecules and secrete inflammatory chemokines to bind and recruit immune cells, respectively, and inflammatory signals increase endothelial permeability to allow for immune infiltration to neighboring tissues^[75]^. Similarly, inducing cellular senescence in VSMCs increases vascular immune cell infiltration^[76]^. Importantly, increased senescent vascular and immune cells manifest in atherosclerotic plaques and exacerbate atherosclerotic pathology via dysregulation of immune responses exerted by the SASP^[77]^.

##### Cell-cell contact

Unfavorable cell-cell contact manifests in senescent vascular cells as increased expression of adhesion molecules and reduced expression of junction proteins^[78,79]^. As previously mentioned, senescent endothelial cells have increased the expression of adhesion molecules on the cell surface, which attracts neighboring cells or cells in the circulation to local senescent cells^[79]^. Despite this improved contact, senescent cells leverage this mechanism to promote the senescent program in their microenvironment rather than for the enhancement of favorable communication^[79]^. In contrast, junction proteins, which are transmembrane proteins that aim to transfer material between cells, are reduced and impair cell-cell communication. Given the key role of endothelial cells in forming barriers to regulate the transport of factors from circulation to peripheral tissues, the dysregulation of junction proteins in senescent vascular cells leads to an impairment of the transport of essential nutrients and oxygen^[78]^. Specifically, dysfunctional barrier function in senescent endothelial cells has been observed in the brain^[80]^, lung^[81]^, and eye^[82]^ and may result in dysregulated permeability of factors into these tissues^[78]^. As such, senescent cells contribute to the established link between increased vascular permeability and vascular dysfunction^[8]^.

##### Oxygen partial pressure

Finally, it is well established that culturing cells at higher oxygen tensions hastens the onset of cellular senescence, and the partial pressure of oxygen is 97%–44%, which is greater in the vascular circulation compared to the interstitial space of other tissues^[72]^. The partial pressure of oxygen varies from blood to tissues to cells, and exposure to higher partial pressures of oxygen *in vivo* likely promotes cellular senescence through the production of ROS^[83,84]^ produced endogenously by vascular cells, or by circulating immune cells, neighboring tissues, or through iatrogenic means.

#### Potential novel mechanisms of vascular senescence

As previously mentioned, the accumulation of senescent cells mediates age-related vascular dysfunction, and clearance of excess senescent cells improves vascular function in old mice^[13,85]^. However, the direct mechanisms by which senescent cells promote vascular aging are not fully understood. In the following section, we describe potentially novel and understudied mechanisms that could regulate cellular senescence-mediated vascular aging [Figure 2]. Although causal evidence may not exist for all these mechanisms contributing to cellular senescence-mediated vascular aging, the processes warrant mention, given that they may provide insight into future experimental considerations.

##### Circadian misalignment

Circadian rhythms are approximately 24-h biological oscillations that have evolved in response to predictable environmental cues^[86]^. All cells, including cells of the vasculature, contain precisely regulated and conserved transcriptional-translational feedback loops, which perpetuate the 24-h oscillations and ultimately regulate fundamental circadian rhythms (also referred to as clock components)^[86]^. *Bmal1* is the only imperative clock gene required for circadian rhythm regulation^[87]^ and is considered the master regulator of the circadian clock^[88]^. Moreover, the BMAL1 protein is a key transcription factor for rhythmic control of chromatin accessibility, thus influencing system-level gene expression^[89]^. Circadian misalignment is associated with early vascular aging in humans^[90]^. The physiological oscillations in *Bmal1* transcription are dampened with aging^[91]^ and knockout of *Bmal1* can directly impair vascular function in young mice^[92]^. Moreover, *in vitro* evidence suggests that dampened *Bmal1* oscillations contribute to the accumulation of senescent cells^[93]^. Thus, circadian misalignment, particularly dampened oscillations in *Bmal1* transcription, may be a novel mechanism underlying cellular senescence-induced vascular aging.

##### Glycolytic byproducts

As mentioned above, mitochondrial dysfunction is a key characteristic of senescent cells. Mitochondrial dysfunction is often accompanied by a compensatory reliance on glycolysis for ATP production^[94]^, and consistent with this concept, senescent cells demonstrate a preferential reliance on glycolysis^[95]^. Considering mitochondrial dysfunction is a well-established biological hallmark of aging^[96]^ and dysfunctional mitochondria are directly implicated in vascular aging^[47]^, it is warranted to investigate the role of increased glycolytic flux as a mechanism mediating cellular senescence-induced vascular aging. Serum proteomic analyses reveal that increased glycolytic metabolism is likely responsible for circulating SASP-mediated changes in vascular aging^[13]^. Indeed, increased glycolytic flux is implicated in vascular aging, due in part to increased production of the glycolytic byproduct and AGE precursor, methylglyoxal (MGO)^[97]^. Under normal physiological conditions, MGO is detoxified via the glyoxalase-1 (GLO-1) pathway; however, GLO-1 activity is reduced with aging, resulting in a greater abundance of MGO^[98]^. In further support of this concept, MGO has been shown to stimulate cellular senescence in human keratinocytes^[99]^ and in the brain^[100]^, and increasing GLO-1 activity has been demonstrated to reduce senescent cell burden in renal cells^[101]^. Moreover, preservation of GLO-1 activity prevents age-related vascular endothelial dysfunction^[102]^. Thus, investigating the role of glycolysis and MGO/GLO-1 balance in mediating cellular senescence-induced vascular aging may be valuable.

##### Membrane contact sites

Changes in membrane contact sites are emerging as biological mechanisms of aging, which has been reviewed in detail elsewhere^[103]^. Membrane contact sites comprise physical bridges that allow for the exchange of metabolites between organelles, ultimately regulating intracellular metabolic flux. Moreover, membrane contact sites create subcellular signaling platforms, which enable local protein modifications and interactions^[103]^. Two organelles that have garnered attention in the context of membrane contact sites are mitochondria and endoplasmic reticulum (ER), referred to herein as mitochondria-ER contacts (MERCs).

MERCs are involved in the regulation of inflammation and redox signaling, and thus have been implicated in age-related pathologies, including adverse vascular remodeling^[103]^. Exposure of endothelial cells in culture to oxidized low-density lipoprotein, an established inducer of cellular senescence^[104]^, results in an upregulation of MERCs^[105]^. Moreover, artificial tightening of MERCs in human fibroblasts leads to the induction of cellular senescence, a heightened proinflammatory secretory profile, and increased ROS production^[106]^. Inversely, the deletion of MERC-related proteins in senescent cells has shown to reduce overall senescence burden and favorably modulate the SASP^[107,108]^. These results suggest there is a clear relation between MERCs and cellular senescence, but the extent to which MERCs mediate cellular senescence (or vice versa) is not well understood. Furthermore, it remains to be determined if MERCs regulate the SASP and how MERCs and cellular senescence and the SASP interact to mediate vascular aging.

##### Endothelial-to-mesenchymal transition

The endothelial-to-mesenchymal transition (EndoMT) is a process by which endothelial cells lose their characteristic markers, such as VE-cadherin, CD31, TIE2, and Von Willebrand factor (VWF), while gaining increased expression of mesenchymal markers, such as alpha 2 smooth muscle actin (α-SMA), fibroblast-specific protein 1 (FSP1), and type I/III collagen, which ultimately leads to a shift toward enhanced contractile properties and reduced NO production^[109]^. EndoMT is commonly provoked by states of excessive inflammation and is highly implicated in vascular aging^[109]^; however, sources of inflammation mediating EndoMT are not fully understood. Senescent endothelial cells are more susceptible to undergoing EndoMT and the proinflammatory SASP may drive the EndoMT in non-senescent endothelial cells^[110]^. An emerging proinflammatory SASP factor thought to promote the endoMT is transforming growth factor-β (TGF-β), as it is a common component of the SASP^[110]^ and an established inducer of the endoMT^[109]^. Although physiological levels of TGF-β are required for homeostatic endothelial function^[111]^, excessive production of TGF-β can promote vascular aging^[112,113]^. As such, the EndoMT may be a mechanism by which cellular senescence and the SASP contribute to vascular aging.

### GROUPS WITH HIGH CELLULAR SENESCENCE BURDEN

**Figure 3.**
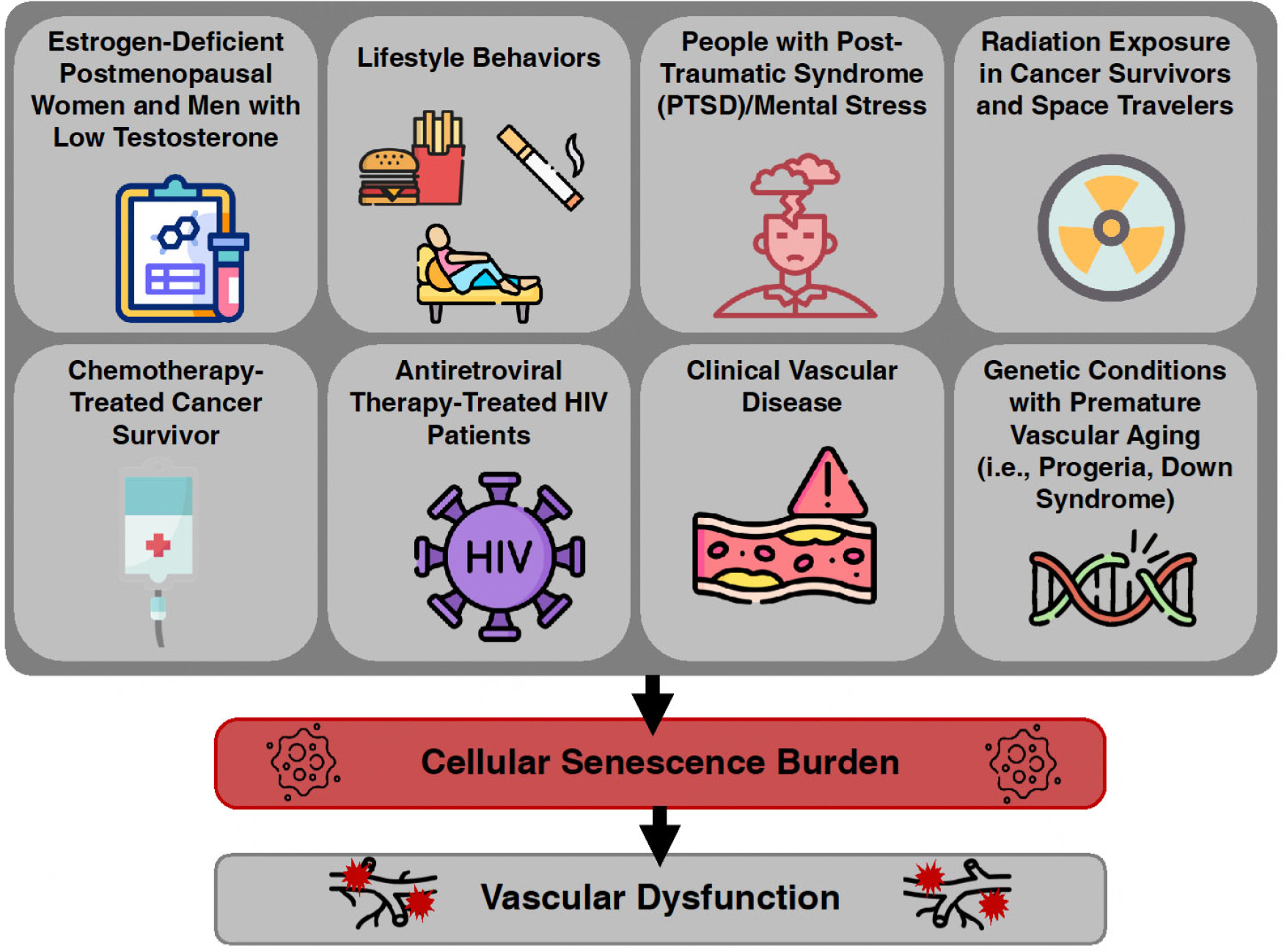
Select groups with high cellular senescence burden and premature vascular aging. Groups that may have high senescent cell burden often experience early vascular aging phenotypes. HIV: Human immunodeficiency virus; CVD: cardiovascular disease.

Cellular senescence increases with chronological age and due to other intrinsic and extrinsic stressors. As such, senescent cells accumulate disproportionally in different individuals and groups and may contribute to vascular health disparities. Conditions of early vascular aging - states in which biological age is greater than chronological age - also often demonstrate increased senescent cell accumulation^[114]^. In this section, we highlight select groups that may experience early vascular aging and a greater senescence burden due to chronological aging, lifestyle choices, genotoxic drugs, genetic mutations, or disease progression^[114]^ [Figure 3].

#### Estrogen-deficient postmenopausal women and men with low testosterone

Aging is characterized in part by a decline in gonadal function, which presents in late life as estrogen deficiency in women and low testosterone levels in men, both of which are risk factors for the development of CVD^[115]^. Vascular function declines in parallel with gonadal aging in women and emerging evidence suggests this may also be the case for older adult men with low testosterone^[116,117]^. However, the mechanisms by which gonadal aging mediates vascular dysfunction are incompletely understood. Physiological levels of estrogen maintain homeostatic redox balance in mitochondria, and as such, estrogen deficiency can increase tonic mitochondrial ROS production. Considering mitochondrial ROS is an established inducer of cellular senescence, it is plausible that vascular aging in estrogen-deficient women is mediated by an increase in cellular senescence. Moreover, in a mouse model with accelerated senescent cell accumulation, estrogen deficiency exacerbates vascular aging^[118]^, demonstrating an interaction between gonadal status, cellular senescence, and vascular aging. Low testosterone levels have long been associated with increased tonic ROS production and chronic low-grade inflammation; therefore, it appears likely that testosterone also regulates cellular senescence. Estrogen- and testosterone-based hormone replacement is contraindicated for CVD prevention for a variety of reasons; thus, rather than replacing hormone levels to improve vascular function in estrogen-deficient postmenopausal women and in men with low testosterone, it may be more beneficial to target the mechanisms (potentially cellular senescence) by which the reductions in these hormones drive vascular aging.

#### Lifestyle behaviors

Certain detrimental lifestyle behaviors may also lead to cellular senescence accumulation and early vascular aging. In preclinical models, a high-fat diet promotes vascular remodeling and stimulates vascular cell senescence^[119]^. This is in agreement with clinical studies observing that exercise-trained mid-life/older adults have less endothelial senescent cell burden than sedentary mid-life/older adults^[42]^. Additional detrimental lifestyle behaviors that have been linked to increased vascular cell senescence include cigarette smoking^[120]^ and poor sleep quality^[121]^. However, more studies are needed to establish the mechanisms and implications of lifestyle behaviors on cellular senescence and early vascular aging and determine if healthy lifestyle behaviors promote healthy vascular aging.

#### Mental stress

Mental stress has emerged as an important risk factor for the development of CVD^[122,123]^ as it promotes early vascular aging phenotypes^[124]^, as reviewed in detail elsewhere^[122,123,125]^. In brief, there are many shared biological processes between the effects of mental stress and the mechanisms underlying age-related vascular dysfunction. Mental stress is associated with reduced NO bioavailability, impaired endothelial function, tonic elevations in ROS, and chronic low-grade inflammation^[124]^, all of which are key mechanisms of vascular aging and features of excess cellular senescence^[11,126]^. Preclinical literature suggests that experimentally provoked mental stress can promote cellular senescence in the brain^[127]^, and patients who experience frequent episodes of mental stress have higher circulating markers of cellular senescence relative to healthy controls^[128]^. Post-traumatic stress disorder (PTSD) is a severe type of mental stress and a psychiatric/mental condition affecting approximately 5% of the US population^[129,130]^. Individuals most affected by PTSD include military combat veterans, victims of assault and abuse, and those who have experienced major disasters^[131]^. PTSD is associated with a reduced overall and healthy lifespan, inferring PTSD accelerates biological aging^[132]^. As such, the relationship between PTSD and the biological mechanisms of aging has gained significant attention over the last decade. Interestingly, a comprehensive review compared SASP markers from numerous studies measuring individual factors in people with PTSD and found multiple lines of investigation suggesting that PTSD may be associated with a proinflammatory SASP profile compared to age-matched individuals without PTSD^[133]^. However, it remains to be determined if PTSD and mental stress directly increase the burden of cellular senescence in the vasculature and if the accumulation of senescent vascular cells is mechanistically implicated in mental stress-induced vascular aging.

#### Radiation

Radiation is a well-established inducer of cellular senescence that occurs in diverse settings and is most commonly experienced by cancer patients via ionizing radiation during their cancer treatment and by space travelers through high solar and cosmic radiation^[134]^. During radiation exposure, high-energy rays induce DNA damage, leading to the induction of cellular senescence, which has been well documented in endothelial cells and VSMCs^[134,135]^. Specifically, exposure of vascular cells to ionizing radiation induces cellular senescence and activates SASP-mediated inflammation^[135]^. In mice, ionizing radiation exposure has been shown to mediate DNA damage-induced vascular cell senescence and inflammation, and accelerated plaque formation in atherosclerotic mice^[136]^. Moreover, cancer survivors who received cancer treatment with ionizing radiation had 2-fold higher arterial stiffness in comparison to cancer patients who did not receive ionizing radiation^[137]^. Together, these data provide evidence that radiation induces vascular cell senescence and promotes early vascular aging.

#### Chemotherapy treatment

Common forms of cancer, including breast cancer, leukemia, and lymphomas, are often treated with anthracycline chemotherapeutic agents^[138,139]^. Anthracyclines are highly effective at treating cancer but are considered to be the most cardiotoxic, as patients treated with anthracyclines experience early CV-related morbidity and often die prematurely from CVD^[138,139]^. A key mechanism by which anthracyclines inhibit cancer growth is through the induction of cellular senescence^[140]^, and cancer survivors previously treated with anthracyclines experience heightened (relative to age- and sex-matched healthy controls) senescent cell burden years into survivorship^[141–143]^. Considering the link between vascular dysfunction and overt CVD, as well as the role of cellular senescence in mediating vascular aging, it is highly plausible that cellular senescence-induced vascular aging is responsible for the early CV-related morbidity and mortality observed in these patients. Doxorubicin, the most commonly administered anthracycline chemotherapeutic agent, is a well-established inducer of cellular senescence and early vascular aging^[144]^. Specifically, young mice that received systemic, genetic clearance of senescent cells following doxorubicin treatment do not develop vascular dysfunction compared to doxorubicin-treated mice that did not receive genetic senescent cell clearance^[145]^. Other classes of chemotherapeutic agents are known to elicit cellular senescence and require further investigation into adverse effects on the vasculature^[146]^. Specifically, paclitaxel, a commonly used microtubule inhibitor, has direct adverse effects on the proliferation and migration of senescent vascular cells and contributes to the disruption of blood-brain barrier integrity^[147,148]^. Together, these data suggest that cellular senescence is likely an underlying mechanism of cancer chemotherapy-induced early vascular aging.

#### Antiretroviral therapy treatment

Although antiretroviral therapies are highly effective at treating the fatal effects of the sexually transmitted human immunodeficiency virus (HIV) infection, long-term use of these therapies promotes early vascular aging and accelerated senescence accumulation^[149]^. Antiretroviral therapies inhibit the reverse transcription utilized by HIV viruses to infect cells^[150]^. Like chemotherapeutic agents, antiretroviral therapies are considered to mediate stress-induced premature senescence^[151]^. Studies in cultured endothelial cells showed that antiretroviral therapies elicit premature senescence associated with inflammation, oxidative stress, and altered eNOS activation^[152]^. Additional preclinical studies in mice demonstrated that antiretroviral therapies promote states of cellular senescence by impairing mitochondrial function, defecting nuclear wall structure, and promoting the SASP, which was accompanied by physiological dysfunction^[151]^. In people, associations have emerged between HIV infection, antiretroviral therapy, and cellular senescence burden^[150,153]^. However, the direct effects of antiretroviral therapies on the vascular function of HIV-infected patients are still yet to be determined.

#### Vascular disease

The presence of clinical vascular disorders and associated risk factors may be another contributor to early vascular aging by increasing cellular senescence. Increased senescent cell burden has been observed in several clinical vascular disorders including atherosclerosis^[154,155]^, pulmonary hypertension^[38,156]^, and aortic aneurysms^[157,158]^; however, it is unclear whether senescent cells are primary or secondary to the onset of these conditions. Given that senescent cell accumulation is only associated with clinical vascular diseases, preclinical models are currently being leveraged to better understand the implications of cellular senescence in these conditions. Below, we highlight studies in which senescent cells play a role in vascular pathologies.

Senescent cells have been identified in atherosclerosis, a progressive disease defined by the accumulation of lipid and fibrous elements in the large elastic arteries^[35]^. Senescent cells exist in atherosclerotic lesions and contribute to the destabilization of plaques that may promote acute CV events^[44,159]^. VSMCs derived from plaques exhibit key senescent cell characteristics, including DNA damage and telomere shortening^[155]^. Further, SASP factors have been shown to aggravate atherosclerosis by recruiting and activating immune responses and promoting chronic inflammation and oxidative stress^[160]^. Pulmonary arterial hypertension is a proliferative disorder that results mainly from increased vascular cell production and contributes to CVD events and chronic lung diseases^[38]^. Cellular senescence contributes to pulmonary vascular remodeling, which ultimately manifests in vascular dysfunction in the lung as signified by increased right ventricular systolic pressure^[38,156]^. Yet, senescent cells may play a multifaceted role in the stability of the pathology by inhibiting the worsening of the pulmonary hypertension state (discussed further in this review)^[38]^. As such, more studies are required to determine the cost-benefit of senescent cell burden in pulmonary arterial hypertension states. Senescent cells in aortic aneurysms, characterized by a persistent, localized dilation (ballooning) of the aorta, contribute to a loss of aortic structural wall integrity, which may lead to severe CVD events^[157,161]^. Proteomic analyses have identified cellular senescence as a key pathway in thoracic aortic aneurysms^[158]^. Senescent cells also contribute to aortic aneurysm development through the secretion of proinflammatory cytokines and ROS through the SASP^[161]^. Senescent cells accumulate primarily at sites of vascular hypertrophy in pulmonary hypertension models^[162]^ and contribute to the pathology through the recruitment of inflammatory cells by the SASP and by promoting vascular cell growth and migration^[38,156]^. Further mechanistic studies are needed to uncover the complex interaction between cellular senescence and vascular disease.

#### Progeria

Hutchinson-Gilford progeria syndrome (HGPS) is an extremely rare, premature aging disorder caused by the genetic mutation of the *Lmna* gene resulting in a defective nuclear lamina^[163]^. In patients with HGPS, there is a greater senescent cell burden and vascular dysfunction that manifests at young ages, with CVD being the leading cause of death in this group^[164]^. Mouse models of progeria have been developed to study disease progression and have also been adapted to study premature aging^[12,165]^. For example, the ERCC1 knockout mouse model depletes the enzyme responsible for multiple DNA repair mechanisms that protect the nuclear genome, resulting in progeria disease progression^[165]^. At young ages, ERCC1 knockout progeroid mice have increased cellular senescence burden in all tissues comparable to old mice, with the aorta having the greatest burden^[12]^. Like HGPS patients, the ERCC1 knockout progeria model exhibits impaired vascular function even at young ages^[166]^. However, more studies are required to determine if these vascular compromises are directly mediated by cellular senescence burden.

Another progeroid syndrome, Werner syndrome, is defined by a mutation in the *Wrn* gene, which encodes the WRN RecQ helicase^[167]^. This helicase has a variety of functions, including DNA replication and repair, telomere maintenance, and epigenetic regulation, such as heterochromatinization^[167]^. Unlike HGPS, Werner syndrome patients develop normally until after puberty, after which many age-related phenotypes manifest, including hair graying, sarcopenia, skin abnormalities, cataracts, atherosclerotic disease, and some cancer, with most patients succumbing to one or more malignancies in their fifth decade of life^[167]^. CVD is a major cause of morbidity and mortality in these patients as they demonstrate dyslipidemia, hypertension, and atherosclerosis at a very early age^[168]^. One of the most profound phenotypes known is the accelerated induction of cellular senescence during replicative culture collected from Werner syndrome patients^[169]^. *In vitro* models suggest that macrophages from Werner syndrome patients co-cultured with endothelial cells and VSMCs are sufficient to induce endothelial cell dysfunction and a synthetic VSMC phenotype^[170]^. Future studies are required to further document the direct contribution of vascular cell senescence in vascular dysfunction and CVD progression in Werner syndrome patients and in preclinical models of the disorder.

#### Down syndrome

Down syndrome is a genetic disorder caused by the presence of all or part of a third copy of chromosome 21 (trisomy 21; +21). It is not only associated with intellectual disability but also with a group of clinical manifestations of premature aging. These include but are not limited to Alzheimer’s disease, cancer, hearing and vision declines, and suppressed immune system function. Like progeria conditions, CVD is prevalent in people with Down syndrome and is the leading cause of death^[171]^. One key insight is that people with Down syndrome have blunted endothelial function compared to age-matched controls^[172]^. Furthermore, initial studies suggest alterations in vascular function may contribute to some of the age-related diseases in this group. Decreased cerebral blood flow has been observed prior to β amyloid accumulation in mouse models of Down syndrome^[173,174]^. Moreover, individuals with Down syndrome demonstrate less carotid blood flow and vascular conductance compared with individuals without Down syndrome, suggesting that alterations in vascular control mechanisms are present in Down syndrome^[175]^. Despite this, little is known about how vascular cell senescence and function are altered with aging in this group. While measures of cellular senescence have not been made in vascular cell types, cellular senescence markers are high in neural progenitor cells^[176]^, primary fibroblasts^[177]^, and primary lymphocytes^[178]^ from people with Down syndrome. Thus, future studies should evaluate vascular cell senescence in people with Down syndrome and further explore if vascular cell senescence is a contributor to vascular dysfunction and elevated CVD risk in people with Down syndrome.

### SENOTHERAPEUTICS AND TRANSLATION TO CLINICAL RESEARCH TO IMPROVE VASCULAR FUNCTION

The select groups discussed above represent groups that experience early vascular aging likely due in part to excess cellular senescence. As such, the groups stand to benefit the greatest from the clearance of excess senescent cells. In the following section, we discuss therapeutic strategies that may be leveraged to reduce vascular cell senescence and the SASP and improve vascular function. Senotherapeutics are a broad class of therapeutic strategies aimed at targeting excess senescent cells and/or the SASP^[179]^. Senotherapeutics often refer to natural or synthetic pharmacological agents but have also been attributed to lifestyle interventions such as aerobic exercise and weight loss in people^[42,179–181]^. There are two main classes of pharmacological senotherapeutics: senolytics and senomorphics^[179]^. Senolytic therapies are interventions that selectively suppress excess senescent cells by targeting SCAP networks^[46]^, whereas senomorphics are compounds that suppress SASP signaling by modulating senescent cell transcription^[182]^. The use of senolytics or senomorphics in pathological states is challenged and highly dependent on the context (discussed in further detail later in the review)^[179]^. Here, we examine evidence of senotherapeutics treatment in vascular studies [Table 1] and discuss the benefits and drawbacks of the senotherapeutic strategies. Although several senolytic therapies are currently being tested in clinical trials, most evidence of senolytic activity on vascular function is reported in cell culture and preclinical models.

#### Senolytics

Select well-established and experimental senolytic therapies exist that suppress senescent cells by targeting SCAP networks to allow apoptosis to resume in senescent cells such that they are cleared from the tissue^[183]^. Senolytics therapies are often administered using a “hit-and-run” approach, which is also often referred to as an intermittent dosing paradigm^[183]^. This approach allows high doses of senolytics to be administered such that excess senescent cell burden can be suppressed back to basal levels. Once senescent cells reaccumulate (~2–4 weeks later in mid-life/older adults), another dosing round is administered. This approach is thought to allow for senescent cells to maintain their physiological roles (i.e., cancer suppression) while keeping levels and pathological effects to a minimum^[11,29,32]^. Using this dosing approach, senolytic therapies have been shown to reduce age-related senescent cell burden and improve vascular function with chronological aging, early aging, and CVD [Table 1].

##### Navitoclax

Initial studies investigating the role of cellular senescence in vascular dysfunction used one of the first senolytics to be established, navitoclax (also known as ABT-267 and ABT-737). Navitoclax is one of the most potent and broad-spectrum senolytic agents to be identified to date^[184]^. However, due to its chemotherapeutic properties, its translation to healthy mid-life/older adults is limited^[185]^. As such, navitoclax is primarily utilized as a “theoretical” senolytic in basic biological and preclinical studies. Preclinical studies have shown that navitoclax-induced senescent cell clearance improves endothelial function and lowers arterial stiffness in old mice^[13]^, but may have detrimental effects on mouse models of vascular pathologies including atherosclerosis^[36,186–188]^ and pulmonary hypertension^[38,156]^.

##### Dasatinib + Quercetin

The combination senolytic treatment, D + Q, is a well-established preclinical intervention that is currently being tested in clinical populations^[189,190]^. Although not related to the vasculature, outcomes from the first completed clinical trials using D + Q have shown that it may reduce cellular senescence markers and select circulating SASP factors in patients with idiopathic pulmonary fibrosis^[189]^ and Alzheimer’s disease^[190]^. In the vasculature, D + Q improved vasomotor function and reduced arterial stiffness in old mice^[85]^. Further, D + Q ameliorated plaques in atherosclerotic mice^[85]^ and abdominal aortic aneurysms in angiotensin II-induced mice^[191]^.

##### Fisetin

Fisetin is a natural flavonoid with senolytic properties that is currently considered to be the safest compound for translation to mid-life/older adults^[192]^. In old mice, intermittent fisetin supplementation improved endothelial function, lowered arterial stiffness, and ameliorated vascular cell senescence burden^[65]^. Several clinical trials are underway testing the effects of fisetin supplementation on chronic conditions, including two studies that are focusing on vascular-related conditions including fisetin supplementation in healthy mid-life/older adults on vascular function (NCT06133634) and in people with peripheral artery disease (NCT06399809).

##### Experimental senolytics

Finally, experimental senolytic interventions, including 25-hydroxycholesterol^[193]^, FOXO4-DRI (a peptide agonist p53 and peroxisomal membrane protein 4 [PXO4] interaction)^[38]^, bis-2-(5-phenylacetamido-1,3,4-thiadiazol-2-yl)ethyl sulfide (BPTES; a glutaminase 1 inhibitor)^[194]^, and a vaccine against glycoprotein nonmetastatic melanoma protein B (GPNMB; a transmembrane protein inhibitor)^[195]^, have demonstrated initial efficacy at reducing senescent cell burden and modulating vascular function or pathological vascular disorders in preclinical models. Preliminary studies of 25-hydroxycholesterol, an endogenous cholesterol metabolite, found that vascular SCAPs were modulated to favorably modulate vascular wall remodeling and reduce arterial stiffening in old mice^[193]^. Notably, FOXO4-DRI worsened CV outcomes in animal models of pulmonary arterial hypertension by promoting unfavorable vascular wall remodeling^[38]^, whereas BPTES and GPNMB interventions both had favorable effects on plaque pathology in atherosclerotic animals^[194,195]^. More testing is required to determine the mechanisms, safety, tolerance, and efficacy of both recognized and experimental senolytic agents in people.

##### Senomorphics

Established senomorphic therapies include statin, antidiabetic, and anti-inflammatory compounds, which all represent natural or FDA-approved drugs^[179]^. Senomorphics target SASP-mediated pathways in senescent cells by modulating central inflammatory regulators, including the transcription factor NF-κB and AKT pathway^[182]^. Though senomorphics are less established than senolytics, there may be contexts in which they are safer and more efficacious depending on pathological conditions (discussed in detail further in the review). Given the limited investigations of these compounds for their senomorphic properties, we will highlight the existing preclinical and cell culture evidence in vascular tissue or cells [Table 1].

##### Statins

Statins have various protective effects on the vasculature, including lowering cholesterol levels. However, certain protective effects may also be attributed to the inhibition of endothelial SASP production^[196]^. For example, pitavastatin inhibits endothelial cell senescence through AKT-dependent mechanisms in both hydrogen peroxide-induced senescence in cultured endothelial cells and streptozotocin-diabetic mice^[196]^.

##### Antidiabetic drugs

Several antidiabetic drugs have been found to have beneficial effects on vascular cell function in addition to their traditional uses on glucose regulation. Of note, it is believed that metformin and glucagon-like peptide 1 (GLP-1) agonists exert some physiological benefits with aging by suppressing SASP signaling. Metformin is a potent suppressor of chronic inflammation that blocks NF-κB activity in doxorubicin-induced senescent endothelial cells^[197]^. Likewise, agonists of the antidiabetic hormone GLP-1, including exendin-4, may mitigate cellular senescence induction and SASP signaling in senescent endothelial cells^[198]^ and VSMCs^[199]^ in culture. In both cell types, exendin-4 inhibits signal transduction system that uses cyclic adenosine monophosphate (cAMP) to activate protein kinase A (PKA)^[198,199]^. As such, antidiabetic drugs may promote their beneficial properties in part through senomorphic effects and may be promising candidates for repurposing to more general aging populations.

##### Anti-inflammatory drugs

Several natural and synthetic anti-inflammatory compounds have capabilities of suppressing circulating SASP factors and modulating SASP signaling in vascular cells. Aspirin is a widely prescribed nonsteroidal anti-inflammatory drug that has also been proposed to have putative senomorphic attributions^[200]^. Endothelial cells in culture have delayed replicative senescence, reduced oxidative stress, and enhanced NO bioavailability following aspirin treatment^[200]^. Resveratrol is a natural polyphenol compound found in the skin of grapes that exerts cardioprotective effects by targeting excess oxidized low-density lipoprotein^[201]^. Resveratrol treatment on replicative senescent endothelial progenitor cells in culture prevents the onset of cellular senescence through activation of AKT pathways^[201]^. Further, VSMCs isolated from old rhesus monkeys have lower SASP production after *in vitro* resveratrol treatment, an effect that is mediated by an inhibition of NF-κB^[202]^. Finally, the immunosuppressor rapamycin and its analogs have been assessed for their senomorphic properties in senescent vascular cells. In old mice, dietary rapamycin supplementation reverses age-related vascular dysfunction while modulating cellular senescence and SASP signaling pathways^[203]^. Rapamycin promotes endoMT during stress-induced premature senescence through the activation of autophagy^[204]^. Together, assessing the senomorphic properties of these anti-inflammatory drugs enables the elucidation of the mechanisms of actions and optimal use of these compounds.

#### Senotherapeutic dosing

**Figure 4.**
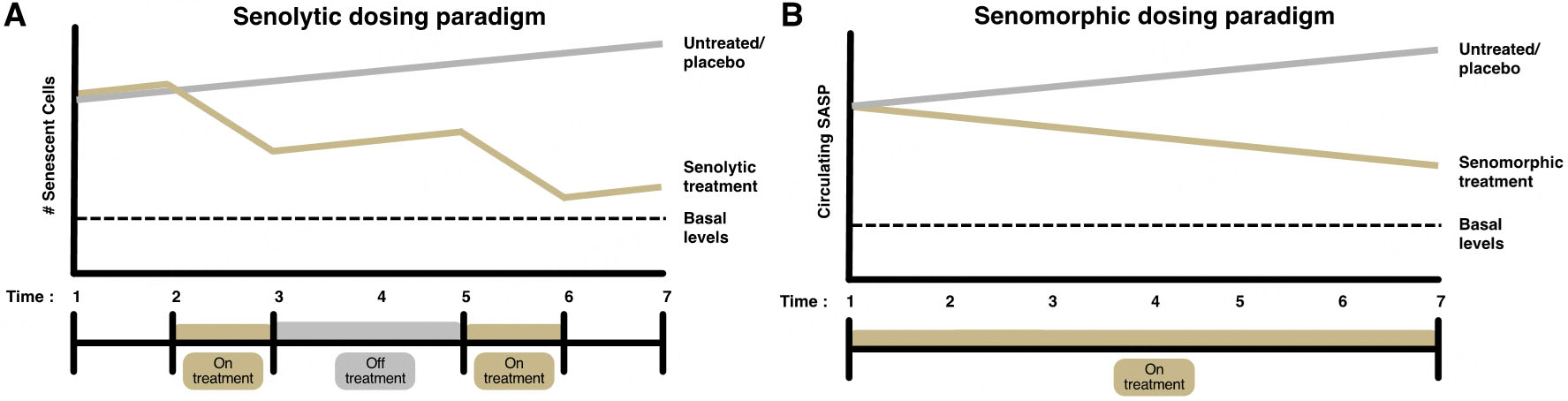
Senotherapeutic dosing paradigms. Senolytic therapies are often administered in an intermittent dosing paradigm composed of 2–6 cycles, each involving 1–7 days of high-dose treatment, followed by a 1–4 week rest period (off treatment), allowing for the suppression of excess cellular senescence without interfering with the basal levels and functions of senescent cells (A). Senomorphic therapies are often administered continuously at a low dose to suppress excess circulating SASP and SASP signaling while maintaining basal inflammation levels (B).

Intermittent dosing of senolytic therapies has shown success in suppressing excess senescent cells while maintaining the basal physiological function of senescent cells (i.e., cancer suppression, wound healing) in preclinical models^[11,29,32]^. However, limited studies have assessed the long-term effects of senolytic treatments and the number of treatment cycles has yet to be optimized. Current clinical trials testing senolytic therapies range from 2 to 6 cycles with generally 1–4 weeks of rest period in between dosing periods^[189]^. This dosing paradigm is also optimal for patient burden, given that medication is only administered on limited days^[183]^. Given the high plasticity of the vasculature (highly adaptable to change given a stimulus), senolytic therapies likely improve vascular function within a short period of time. However, tissues with less plasticity, such as the heart and skeletal muscle, may require longer dosing periods to observe improvements. Thus, future clinical trials should consider senolytic dosing paradigms based on clinical and functional outcomes [Figure 4].

Senomorphics, on the other hand, may require continuous, low-dose administration, given the constant production of the SASP. However, more preclinical studies are required to elucidate the ideal dosing paradigm^[179]^. Further, given the diversity of the SASP and SASP regulators, multiple senomorphic agents may need to be administered for global SASP suppression^[182]^ [Figure 4]. Finally, more studies are required to compare senolytic versus senomorphic therapies and to determine the context in which each is more optimal.

#### Senotherapeutic intervention considerations in vascular disease

Although it is generally agreed upon that senescent cells contribute to several vascular diseases, senescent cell clearance is contended in select vascular pathologies and disease progression. For example, several studies have investigated the contributions and effects of the elimination of senescent cells in animal models of atherosclerosis. Although several studies found favorable effects of senolytic treatment on atherosclerotic plaques^[36,85,188,194,195]^, two studies found that senescent cell clearance worsened outcomes, including plaque instability and rupture in advanced stages of atherosclerosis^[186,187]^. As such, it is hypothesized that senescent cells may contribute to atherosclerotic plaque structure and more strategies are required to understand the optimal timing for senescent cell clearance. Similar controversies exist in models of pulmonary arterial hypertension. A study by van der Feen *et al.* found that senescent cell clearance in a rat model of pulmonary hypertension reversed disease progression by improving vascular hemodynamics^[156]^. Conversely, Born *et al.* found that senolytics in mice and rat models of pulmonary hypertension aggravated disease severity and worsened vascular hemodynamics^[38]^.

Finally, several senolytic compounds may have adverse safety profiles that limit their translatability to the clinic. In particular, thrombocytopenia, a condition characterized by low circulating platelet count, has been observed with Navitoclax administration^[205]^ and D + Q administration has been reported to have several side effects including hematologic dysfunction, fluid retention and QT prolongation, and state of the extended electrical interval between the heart’s contraction and relaxation^[206]^. These findings collectively suggest that senescent cell clearance has variable effects on vascular disease pathogenesis, and more studies are required to fully elucidate the benefits and limitations of senotherapeutic interventions in these conditions. Further, senomorphic agents may be more efficacious in contexts where senescent cell elimination is detrimental and the development of novel senotherapeutic strategies that have more specific targets may be warranted.

### MULTI-ORGAN BENEFIT OF TARGETING SENESCENCE IN THE VASCULATURE

**Figure 5.**
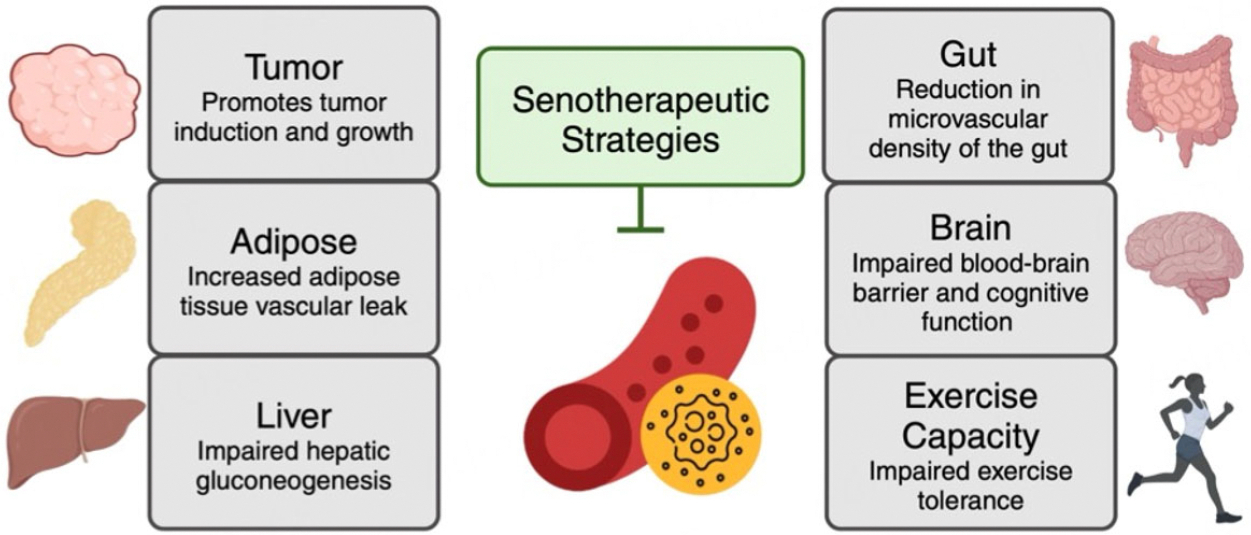
Multi-organ benefit of targeting senescence in the vasculature. Mechanistic studies in preclinical models elucidate that the vascular senescent cells have systemic detrimental effects and clearance of senescent vascular cells elicits improvements in several tissues/organs and physiological processes.

The importance of cellular senescence in the vasculature extends beyond its role in CVD. This is because cellular senescence occurs within vascular cells resident to many tissues and organs and affects neighboring non-vascular cells by disrupting blood flow regulation which is crucial for broader physiological implications [Figure 5]. Thus, studies on vascular cell senescence have been essential to our understanding of the physiological implications of cellular senescence. An increasing number of reports have identified the cells of the vasculature as some of the main cell types that becomes senescent with advancing age as well as in models of genetically and environmentally early aging. Indeed, in these settings, senescent vascular cells arise within nearly all tissues, including the eye^[207,208]^, skeletal muscle^[209]^, heart^[210]^, kidney^[211,212]^, liver^[213–215]^, pancreas^[213]^, testes^[216]^, brain^[217–220]^, adipose tissue^[210,221]^, and lung^[156,222,223]^. In addition to being widespread, endothelial cells are one of the first cell types to become senescent with advancing age^[214]^, and the cellular senescence markers p16 and p21 increase in the vasculature to a greater extent than any other tissue in both natural aging and progeroid mouse models^[12]^. Cumulatively, these findings support a model by which many diseases, as well as premature and healthy aging, result in vascular cell senescence that occurs early, is robustly induced, and is found within nearly every tissue. As such, an additional important benefit of targeting cellular senescence in the vasculature is that it is likely to have systemic benefits. Vascular cellular senescence directly contributes to a host of cardiometabolic diseases and cancer (reviewed in detail elsewhere^[72]^). Recent evidence has shed light on the mechanisms by which vascular senescence contributes to this multi-system dysfunction. This includes (1) induction of cellular senescence in neighboring cells and tissues, as well as an increase in local inflammation; and (2) dysregulation of blood flow. Through these mechanisms, inducing cellular senescence in the vasculature appears to act as a rheostat for the physiological age of an organism. This has been demonstrated by the manipulation of several known modulators of cellular senescence and the SASP in the vasculature.

Several examples of this phenomenon come from studies using genetically modified mice to specifically manipulate endothelial cells. For example, three independent studies have demonstrated the effects of genetic induction of telomere dysfunction specifically within endothelial cells^[40,224,225]^. In endothelial cells, telomere dysfunction stimulated cellular senescence and increased expression of several SASP factors^[40,224,225]^. Consequently, cellular senescence and the SASP are elevated in whole artery lysates, perigonadal white adipose tissue, and the liver^[40,224,225]^. By reducing the replicative capacity of endothelial cells, microvascular density is reduced in the gut^[40]^ and adipose tissue vascular leak is increased^[224]^. Moreover, insulin-mediated production of NO is impaired^[40]^. In these mice, adipose tissue and the liver display elevated markers of senescence and inflammatory SASP factors and impaired glucose tolerance^[40,224,225]^. In addition to the inflammation in metabolically active tissues, alterations in glucose homeostasis may be a consequence of improper nutrient delivery due to a lack of vascularization and blood flow reallocation in response to metabolic stimuli^[40,224,225]^. Finally, running endurance and cognitive function are impaired by endothelial telomere dysfunction-induced senescence^[224]^. These data indicate that stimulating cellular senescence in young mice results in a premature aging phenotype that extends beyond changes typically associated with vascular dysfunction.

Mirroring the studies of telomere dysfunction in young mice, endothelial cell-specific reductions in the mammalian target of rapamycin (mTOR) reduce cellular senescence in both whole arteries and endothelial cells, as well as in metabolically active tissues including the adipose tissue and the liver^[226]^. Likewise, inflammatory SASP factors are reduced in these tissues^[226]^. These changes culminate in improvements in glucose tolerance due to a suppression of hepatic gluconeogenesis^[226]^. Importantly, when coupled with findings that show senescent cell clearance in old mice improves vascular function in a multitude of ways^[13,65]^, these findings demonstrate the multiplicity of benefits of targeting cellular senescence specifically in the vasculature. It appears that senescent cells in the vasculature may promote cellular senescence and inflammation in a variety of tissues, reduce vessel density, increase vascular permeability, and disrupt proper redistribution of blood in response to metabolic stimuli, all of which act to impair metabolic function and potentially exercise tolerance and cognition. Therefore, clearing senescent vascular cells is likely to be beneficial in conditions that were not originally considered to be driven by cellular senescence in the vasculature.

### RESEARCH GAPS AND FUTURE DIRECTIONS

**Figure 6.**
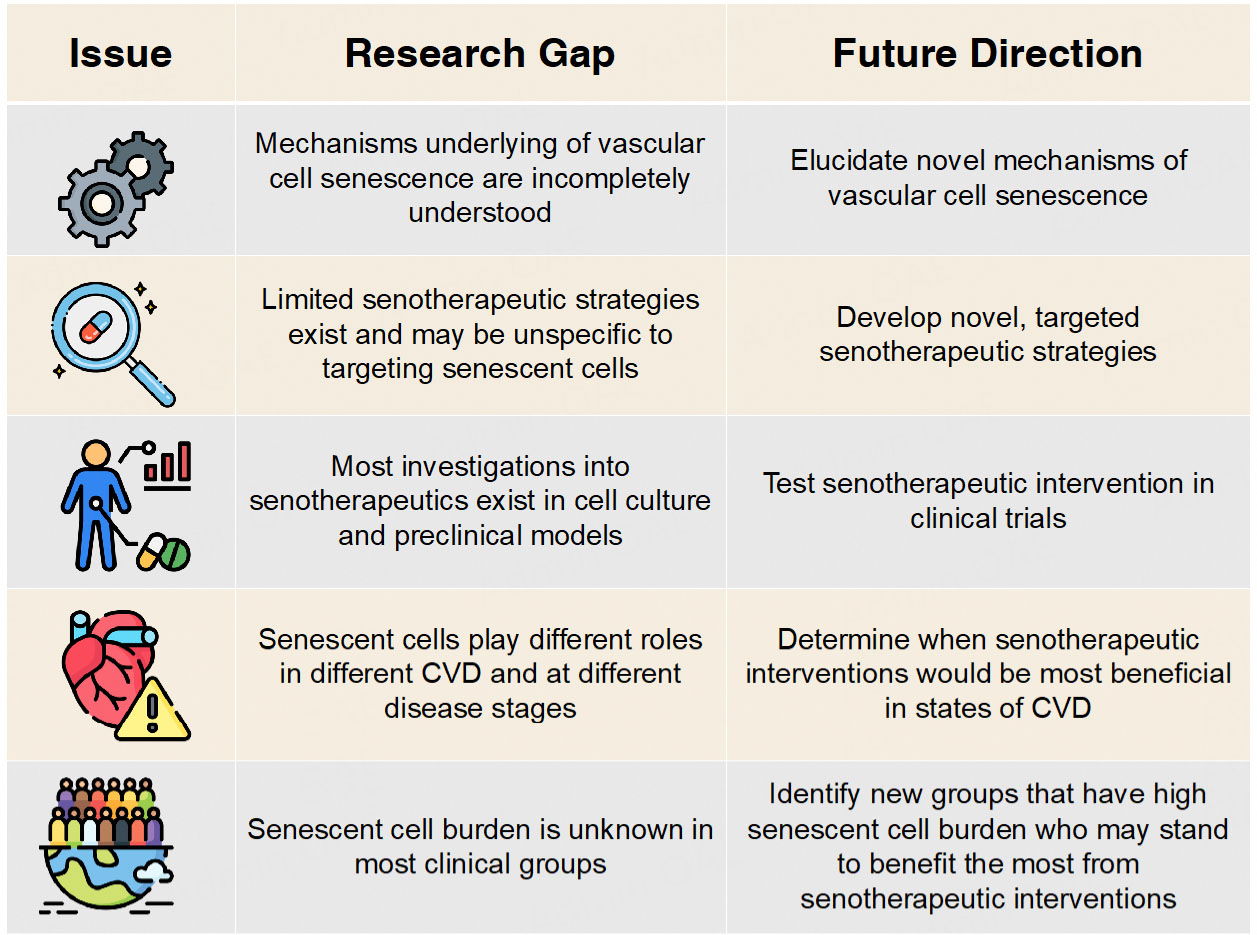
Research gaps and future directions. Presented are important research gaps and future directions in the understanding of cellular senescence, vascular function, and cardiovascular disease (CVD). These research gaps require prioritization in ongoing and future studies.

The role of cellular senescence in vascular aging is an emerging field with promising potential in modulating clinical CV outcomes. Despite the promising evidence in the field, several research gaps exist that represent future directions in CV and cellular senescence research [Figure 6]. At a fundamental level, the mechanisms underlying vascular cell senescence and mechanisms by which senescent cells contribute to vascular dysfunction are yet to be fully elucidated. Uncovering these mechanisms may reveal novel means to target senescent cells and elucidate the detrimental effects of cellular senescence on vascular function. Next, markers of cellular senescence have only recently allowed researchers to assess cellular senescence levels in clinical populations^[31,45]^. As such, senescent cell burden has yet to be characterized in many clinical populations but could be a promising therapeutic target for groups that present with high levels of senescent cells. Even in the pathologies in which senescent cells have been identified, the role of the senescent cells appears to be largely heterogenous, and clearance of senescent cells has variable outcomes. Accordingly, further research exploring and optimizing the dosing, timing, and context of senotherapeutic interventions is essential for translation. Finally, several senotherapeutic strategies exist, but the safety, tolerability, and efficacy of these interventions in people are not fully known^[183]^. Clinical trials of the senotherapeutic compounds discussed in this review are essential, as well as the identification of novel strategies that may target specific cell types or affected areas.

## CONCLUSIONS

Aging is the major risk factor for CVD due to the development of vascular dysfunction, particularly arterial stiffening and endothelial dysfunction. In this review, we discussed cellular senescence - a stress response characterized by cell cycle arrest - as a fundamental mechanism of vascular aging that may serve as viable therapeutic targets for the prevention and treatment of vascular dysfunction to reduce the risk of CVD. We reviewed key established and novel mechanisms by which senescent cells contribute to vascular dysfunction. We identified groups that may have high vascular cell senescence burden due to chronological or early vascular aging. Finally, we discussed senotherapeutics - therapies that suppress excess senescent cells or the SASP - and examined their translational potential, dosing paradigm, clinical implications, groups that may stand the most to benefit, and systemic benefits. Together, this review underscores the crucial role of cellular senescence in vascular dysfunction and the need for deeper understanding of the translational use of senotherapeutics in groups with high senescent cell burden.

## Figures and Tables

**Table 1.** Investigations of senotherapeutic agents in vascular studies

Senotherapeutic (class)	Dosing paradigm	Model	Condition	Senescence/SASP outcome	Vascular outcome	Reference

Navitoclax (senolytic)	50 mg/kg/day 1 week on - 2 weeks off - 1 week on	C57Bl/6 mice	Old age	↓ p16	↑ Endothelial function ↓ Arterial stiffness	Clayton *et al.*, 2023^[13]^
Navitoclax (senolytic)	100 mg/kg/day for 6 weeks or 50 mg/kg/day for 9 weeks	Apoe KO mice	Atherosclerosis	↓ *Cdkn2a* ↓ p21	↓ Plaques stability	Karnewar *et al.*, 2023^[186]^
Navitoclax (senolytic)	50 mg/kg/day 1 week on - 3 weeks off for 3 cycles	p16-3MR Apoe KO mice	Atherosclerosis	No change *Cdkn2a* or SASP	↓ Atherosclerotic plaques	Garrido *et al.*, 2022^[187]^
Navitoclax (senolytic)	100 mg/kg/day for 9 days	p16-3MR Ldlr KO mice	Atherosclerosis	↓ SA-β-Gal and *Cdkn2a*	↓ Atherosclerotic plaques	Childs *et al.*, 2016^[36]^
Navitoclax (senolytic)	100 mg/kg/day 1 week on - 3 weeks off for 3 cycles	C57Bl/6 and INK-ATTAC Ldlr KO mice	Atherosclerosis	↓ SA-β-Gal	↓ Atherosclerotic plaques	Childs *et al.*, 2021^[188]^
Navitoclax (senolytic)	10 mg/kg/day for 8 days	Monocrotaline-treated Wistar rats	Pulmonary arterial hypertension	↓ p16 and p21	↓ RVSP	van der Feen *et al.* 2020^[156]^
Navitoclax (senolytic)	10-50 mg/kg/day for 3 weeks	C57Bl/6 and SM22-5-HTT^+^ mice and Wistar rats	Pulmonary arterial hypertension	↓ p16 and p21 ↓ DNA damage	↑ Vascular remodeling ↓ RVSP	Born *et al.*, 2022^[38]^
D + Q (senolytic)	5 mg/kg/day (D) + 10 mg/kg/day (Q) once weekly for 2 months	C57BL/6 Apoe KO mice	Old age and atherosclerosis	↓ DNA damage	↑ Endothelial function ↓ Arterial stiffness ↓ Atherosclerotic plaques	Roos *et al.*, 2016^[85]^
D + Q (senolytic)	5 mg/kg/day D + 50 mg/kg/day Q 3 days on - 2 weeks off for 3 cycles	C57Bl/6 angiotensin II-induced mice	Old age and abdominal aortic aneurysm	↓ *Cdkn1a* and SASP	↓ Abdominal aortic aneurysm	Parvizi *et al.*, 2021^[191]^
Fisetin (senolytic)	100 mg/kg/day 1 week on - 2 weeks off - 1 week on	C57Bl/6 and p16-3MR mice	Old age	↓ p16, *Cdkn2a* and SCAP	↑ Endothelial function ↓ Arterial stiffness	Mahoney *et al.*, 2024^[65]^
25-Hydroxycholersterol (senolytic)	50 mg/kg/day for 5 days	p16-3MR mice	Old age	↓ p16, *Cdkn2a* and SASP	↓ Arterial stiffness	Mahoney *et al.*, 2023^[193]^
FOXO4-DRI (senolytic)	5 mg/kg/day 3X per week for 3 weeks	C57Bl/6 and SM22-5-HTT^+^ mice	Pulmonary arterial hypertension	None reported	↓ RVSP	Born *et al.*, 2022^[38]^
BPTES (senolytic)	5 g/kg/day 3X per week for 1 month	C57Bl/6 Apoe KO mice	Old age and atherosclerosis	↓ SA-β-Gal and SASP	↓ Atherosclerotic plaques	Johmura *et al.*, 2026^[194]^
GPNMB vaccine (senolytic)	Not reported	C57Bl/6 Apoe KO mice	Atherosclerosis	↓ *Cdkn1a, Cdkn2a*, and SA-β-Gal	↓ Atherosclerotic plaques	Suda *et al.*, 2022^[195]^
Pitavastatin (senomorphic)	10-100 nmol/L once	HUVEC	H_2_O_2_-induced cellular senescence	↓ SA-β-Gal	↑ eNOS, SIRT1, and catalase expression	Ota *et al.*, 2010^[196]^
Pitavastatin (senomorphic)	3 mg/kg/day daily for entire life	Streptozotocin-treated C57Bl/6 mice	Diabetes	↓ SA-β-Gal	↑ eNOS, SIRT1, and catalase expression	Ota *et al.*, 2010^[196]^
Metformin (senomorphic)	0.5-5 mM for 24 h	HUVEC	Doxorubicin-induced senescence	↓ SASP secretion	↓ Endothelial adhesion molecules	Abdelgawad *et al.* 2023^[197]^
Exendin-4 (senomorphic)	10 nmol/l for 72 h	HUVEC	H_2_O_2_-induced senescence	↓ SA-β-Gal activity	None reported	Oeseburg *et al.* 2010^[198]^
Exendin-4 (senomorphic)	0.01-100 nM for 72 h	Rat aortic VSMC	Angiotensin II-induced senescence	↓ SA-β-Gal activity	↓ ROS levels	Xhao et at. 2014^[199]^
Aspirin (senomorphic)	100 M every 48 h until the 12th passage	HUVEC	Replicative senescence	↓ SA-β-Gal activity ↑ Telomerase activity	↓ ROS levels ↑ NO levels	Bode-Böger *et al.*, 2005^[200]^
Resveratrol (senomorphic)	10-50 M for 72 h	HEPC	Replicative senescence	↓ SA-β-Gal activity ↑ Telomerase activity	↑ Cell proliferation and migration	Xia *et al.*, 2008^[201]^
Resveratrol (senomorphic)	1 mol/L for 48 h	Rhesus monkey VSMC	Old age	↓ Cytokine profile ↓ SASP profile	↓ Mitochondrial ROS production	Csiszar *et al.*, 2011^[202]^
Metformin (senomorphic)	0.5-5 mM for 24h	HUVEC	Doxorubicin-induced senescence	↓ SASP secretion	↓ Endothelial adhesion molecules	Abdelgawad *et al.* 2023^[197]^
Rapamycin (senomorphic)	14 mg kg^−1^ for 6 weeks	B6D2F1 mice	Old age	↓ p19	↑ Endothelial function and ↓ Arterial stiffness	Lesniewski *et al.*, 2017^[203]^
Rapamycin (senomorphic)	10 nM for 6, 24, 48, or 72h	HCAEC	H_2_O_2_-induced senescence	↓ SASP secretion ↓ SA-β-Gal	↑ EndoMT transition	Sasaki *et al.*, 2020^[204]^

SASP: Senescence-associated secretory phenotype; KO: knockout; HUVEC: human umbilical vein endothelial cell; HEPC: human endothelial progenitor cell; VSMC: vascular smooth muscle cell; HCAEC: human coronary artery endothelial cell; H_2_O_2_: hydrogen peroxide; SA-β-Gal: senescence-associated β-galactosidase; SCAP: senescent cell anti-apoptotic pathways; BPTES: bis-2-(5-phenylacetamido-1,3,4-thiadiazol-2-yl) ethyl sulfide; GPNMB: glycoprotein nonmetastatic melanoma protein B; RVSP: right ventricular systolic pressure. eNOS: endothelial nitric oxide synthase; ROS: reactive oxygen species; NO: nitric oxide; EndoMT: endothelial-to-mesenchymal transition.

## Data Availability

Not applicable.

## References

[1] Kazi DS, Elkind MSV, Deutsch A, Forecasting the economic burden of cardiovascular disease and stroke in the United States through 2050: a presidential advisory from the American heart association. Circulation. 2024;150:e89–101. 38832515 10.1161/CIR.0000000000001258

[2] Martin SS, Aday AW, Almarzooq ZI, 2024 heart disease and stroke statistics: a report of US and global data from the American heart association. Circulation. 2024;149:e347–913. 38264914 10.1161/CIR.0000000000001209 PMC12146881

[3] Forouzanfar MH, Alexander L, Anderson HR, Global, regional, and national comparative risk assessment of 79 behavioural, environmental and occupational, and metabolic risks or clusters of risks in 188 countries, 1990–2013: a systematic analysis for the Global Burden of Disease Study 2013. Lancet. 2015;386:2287–323. 26364544 10.1016/S0140-6736(15)00128-2 PMC4685753

[4] Olshansky SJ, Goldman DP, Zheng Y, Rowe JW. Aging in America in the twenty-first century: demographic forecasts from the MacArthur Foundation Research Network on an Aging Society. Milbank Q. 2009;87:842–62. 20021588 10.1111/j.1468-0009.2009.00581.x PMC2888016

[5] Lakatta EG, Levy D. Arterial and cardiac aging: major shareholders in cardiovascular disease enterprises: part I: aging arteries: a “set up” for vascular disease. Circulation. 2003;107:139–46. 12515756 10.1161/01.cir.0000048892.83521.58

[6] Seals DR, Kaplon RE, Gioscia-Ryan RA, LaRocca TJ. You’re only as old as your arteries: translational strategies for preserving vascular endothelial function with aging. Physiology. 2014;29:250–64. 24985329 10.1152/physiol.00059.2013 PMC4103060

[7] Abdellatif M, Rainer PP, Sedej S, Kroemer G. Hallmarks of cardiovascular ageing. Nat Rev Cardiol. 2023;20:754–77. 37193857 10.1038/s41569-023-00881-3

[8] Ungvari Z, Tarantini S, Donato AJ, Galvan V, Csiszar A. Mechanisms of vascular aging. Circ Res. 2018;123:849–67. 30355080 10.1161/CIRCRESAHA.118.311378 PMC6248882

[9] Fleenor BS, Seals DR, Zigler ML, Sindler AL. Superoxide-lowering therapy with TEMPOL reverses arterial dysfunction with aging in mice. Aging Cell. 2012;11:269–76. 22168264 10.1111/j.1474-9726.2011.00783.x PMC3409251

[10] Lesniewski LA, Durrant JR, Connell ML, Folian BJ, Donato AJ, Seals DR. Salicylate treatment improves age-associated vascular endothelial dysfunction: potential role of nuclear factor κB and forkhead box O phosphorylation. J Gerontol A Biol Sci Med Sci. 2011;66:409–18. 21303813 10.1093/gerona/glq233 PMC3055281

[11] Campisi J, d’Adda di Fagagna F. Cellular senescence: when bad things happen to good cells. Nat Rev Mol Cell Biol. 2007;8:729–40. 17667954 10.1038/nrm2233

[12] Yousefzadeh MJ, Zhao J, Bukata C, Tissue specificity of senescent cell accumulation during physiologic and accelerated aging of mice. Aging Cell. 2020;19:e13094. 31981461 10.1111/acel.13094 PMC7059165

[13] Clayton ZS, Rossman MJ, Mahoney SA, Cellular senescence contributes to large elastic artery stiffening and endothelial dysfunction with aging: amelioration with senolytic treatment. Hypertension. 2023;80:2072–87. 37593877 10.1161/HYPERTENSIONAHA.123.21392 PMC10530538

[14] Chen MS, Lee RT, Garbern JC. Senescence mechanisms and targets in the heart. Cardiovasc Res. 2022;118:1173–87. 33963378 10.1093/cvr/cvab161 PMC8953446

[15] Green DJ, Jones H, Thijssen D, Cable NT, Atkinson G. Flow-mediated dilation and cardiovascular event prediction: does nitric oxide matter? Hypertension. 2011;57:363–9. 21263128 10.1161/HYPERTENSIONAHA.110.167015

[16] Mitchell GF, Hwang SJ, Vasan RS, Arterial stiffness and cardiovascular events: the Framingham Heart Study. Circulation. 2010;121:505–11. 20083680 10.1161/CIRCULATIONAHA.109.886655 PMC2836717

[17] Chirinos JA, Segers P, Hughes T, Townsend R. Large-artery stiffness in health and disease: JACC state-of-the-art review. J Am Coll Cardiol. 2019;74:1237–63. 31466622 10.1016/j.jacc.2019.07.012 PMC6719727

[18] Owens GK, Kumar MS, Wamhoff BR. Molecular regulation of vascular smooth muscle cell differentiation in development and disease. Physiol Rev. 2004;84:767–801. 15269336 10.1152/physrev.00041.2003

[19] Townsend RR, Wilkinson IB, Schiffrin EL, Recommendations for improving and standardizing vascular research on arterial stiffness: a scientific statement from the American Heart Association. Hypertension. 2015;66:698–722. 26160955 10.1161/HYP.0000000000000033 PMC4587661

[20] Seals DR, Jablonski KL, Donato AJ. Aging and vascular endothelial function in humans. Clin Sci. 2011;120:357–75. 10.1042/CS20100476 PMC3482987 21244363

[21] Cahill PA, Redmond EM. Vascular endothelium - gatekeeper of vessel health. Atherosclerosis. 2016;248:97–109. 26994427 10.1016/j.atherosclerosis.2016.03.007 PMC6478391

[22] Harrison DG. Cellular and molecular mechanisms of endothelial cell dysfunction. J Clin Invest. 1997;100:2153–7. 9410891 10.1172/JCI119751 PMC508409

[23] Steven S, Frenis K, Oelze M, Vascular inflammation and oxidative stress: major triggers for cardiovascular disease. Oxid Med Cell Longev. 2019;2019:7092151. 31341533 10.1155/2019/7092151 PMC6612399

[24] Fukai T, Ushio-Fukai M. Superoxide dismutases: role in redox signaling, vascular function, and diseases. Antioxid Redox Signal. 2011;15:1583–606. 21473702 10.1089/ars.2011.3999 PMC3151424

[25] van der Loo B, Labugger R, Skepper JN, Enhanced peroxynitrite formation is associated with vascular aging. J Exp Med. 2000;192:1731–44. 11120770 10.1084/jem.192.12.1731 PMC2213492

[26] Hsieh HJ, Liu CA, Huang B, Tseng AHH, Wang DL. Shear-induced endothelial mechanotransduction: the interplay between reactive oxygen species (ROS) and nitric oxide (NO) and the pathophysiological implications. J Biomed Sci. 2014;21:3. 24410814 10.1186/1423-0127-21-3 PMC3898375

[27] Faleeva M, Ahmad S, Theofilatos K, Sox9 accelerates vascular aging by regulating extracellular matrix composition and stiffness. Circ Res. 2024;134:307–24. 38179698 10.1161/CIRCRESAHA.123.323365 PMC10826924

[28] Bellien J, Favre J, Iacob M, Arterial stiffness is regulated by nitric oxide and endothelium-derived hyperpolarizing factor during changes in blood flow in humans. Hypertension. 2010;55:674–80. 20083732 10.1161/HYPERTENSIONAHA.109.142190

[29] Aging Campisi J., cellular senescence, and cancer. Annu Rev Physiol. 2013;75:685–705. 23140366 10.1146/annurev-physiol-030212-183653 PMC4166529

[30] Coppé JP, Desprez PY, Krtolica A, Campisi J. The senescence-associated secretory phenotype: the dark side of tumor suppression. Annu Rev Pathol. 2010;5:99–118. 20078217 10.1146/annurev-pathol-121808-102144 PMC4166495

[31] González-Gualda E, Baker AG, Fruk L, Muñoz-Espín D. A guide to assessing cellular senescence in vitro and in vivo. FEBS J. 2021;288:56–80. 32961620 10.1111/febs.15570

[32] Demaria M, Ohtani N, Youssef SA, An essential role for senescent cells in optimal wound healing through secretion of PDGF-AA. Dev Cell. 2014;31:722–33. 25499914 10.1016/j.devcel.2014.11.012 PMC4349629

[33] Baker DJ, Wijshake T, Tchkonia T, Clearance of p16^INK4a^-positive senescent cells delays ageing-associated disorders. Nature. 2011;479:232–6. 22048312 10.1038/nature10600 PMC3468323

[34] Han Y, Kim SY. Endothelial senescence in vascular diseases: current understanding and future opportunities in senotherapeutics. Exp Mol Med. 2023;55:1–12. 36599934 10.1038/s12276-022-00906-w PMC9898542

[35] Hu C, Zhang X, Teng T, Ma ZG, Tang QZ. Cellular senescence in cardiovascular diseases: a systematic review. Aging Dis. 2022;13:103–28. 35111365 10.14336/AD.2021.0927 PMC8782554

[36] Childs BG, Baker DJ, Wijshake T, Conover CA, Campisi J, van Deursen JM. Senescent intimal foam cells are deleterious at all stages of atherosclerosis. Science. 2016;354:472–7. 27789842 10.1126/science.aaf6659 PMC5112585

[37] Cho JH, Kim EC, Son Y, CD9 induces cellular senescence and aggravates atherosclerotic plaque formation. Cell Death Differ. 2020;27:2681–96. 32346137 10.1038/s41418-020-0537-9 PMC7429960

[38] Born E, Lipskaia L, Breau M, Eliminating senescent cells can promote pulmonary hypertension development and progression. Circulation. 2023;147:650–66. 36515093 10.1161/CIRCULATIONAHA.122.058794

[39] Gao P, Gao P, Zhao J, MKL1 cooperates with p38MAPK to promote vascular senescence, inflammation, and abdominal aortic aneurysm. Redox Biol. 2021;41:101903. 33667992 10.1016/j.redox.2021.101903 PMC7937568

[40] Bloom SI, Liu Y, Tucker JR, Endothelial cell telomere dysfunction induces senescence and results in vascular and metabolic impairments. Aging Cell. 2023;22:e13875. 37259606 10.1111/acel.13875 PMC10410008

[41] Jia G, Aroor AR, Jia C, Sowers JR. Endothelial cell senescence in aging-related vascular dysfunction. Biochim Biophys Acta Mol Basis Dis. 2019;1865:1802–9. 31109450 10.1016/j.bbadis.2018.08.008

[42] Rossman MJ, Kaplon RE, Hill SD, Endothelial cell senescence with aging in healthy humans: prevention by habitual exercise and relation to vascular endothelial function. Am J Physiol Heart Circ Physiol. 2017;313:H890–5. 28971843 10.1152/ajpheart.00416.2017 PMC5792201

[43] Chi C, Li DJ, Jiang YJ, Vascular smooth muscle cell senescence and age-related diseases: state of the art. Biochim Biophys Acta Mol Basis Dis. 2019;1865:1810–21. 31109451 10.1016/j.bbadis.2018.08.015

[44] Katsuumi G, Shimizu I, Yoshida Y, Minamino T. Vascular senescence in cardiovascular and metabolic diseases. Front Cardiovasc Med. 2018;5:18. 29556500 10.3389/fcvm.2018.00018 PMC5845435

[45] Dimri GP, Lee X, Basile G, A biomarker that identifies senescent human cells in culture and in aging skin in vivo. Proc Natl Acad Sci USA. 1995;92:9363–7. 7568133 10.1073/pnas.92.20.9363 PMC40985

[46] Zhu Y, Tchkonia T, Pirtskhalava T, The Achilles’ heel of senescent cells: from transcriptome to senolytic drugs. Aging Cell. 2015;14:644–58. 25754370 10.1111/acel.12344 PMC4531078

[47] Rossman MJ, Gioscia-Ryan RA, Clayton ZS, Murphy MP, Seals DR. Targeting mitochondrial fitness as a strategy for healthy vascular aging. Clin Sci. 2020;134:1491–519. 10.1042/CS20190559 32584404

[48] Miwa S, Kashyap S, Chini E, von Zglinicki T. Mitochondrial dysfunction in cell senescence and aging. J Clin Invest. 2022;132:e158447. 35775483 10.1172/JCI158447 PMC9246372

[49] Wiley CD, Velarde MC, Lecot P, Mitochondrial dysfunction induces senescence with a distinct secretory phenotype. Cell Metab. 2016;23:303–14. 26686024 10.1016/j.cmet.2015.11.011 PMC4749409

[50] Victorelli S, Salmonowicz H, Chapman J, Apoptotic stress causes mtDNA release during senescence and drives the SASP. Nature. 2023;622:627–36. 37821702 10.1038/s41586-023-06621-4 PMC10584674

[51] Mahoney SA, Dey AK, Basisty N, Herman AB. Identification and functional analysis of senescent cells in the cardiovascular system using omics approaches. Am J Physiol Heart Circ Physiol. 2023;325:H1039–58. 37656130 10.1152/ajpheart.00352.2023 PMC10908411

[52] Erusalimsky JD. Vascular endothelial senescence: from mechanisms to pathophysiology. J Appl Physiol (1985). 2009;106:326–32. 19036896 10.1152/japplphysiol.91353.2008 PMC2636933

[53] Nguyen MT, Vryer R, Ranganathan S, Telomere length and vascular phenotypes in a population-based cohort of children and midlife adults. J Am Heart Assoc. 2019;8:e012707. 31140354 10.1161/JAHA.119.012707 PMC6585377

[54] Chiu JJ, Chien S. Effects of disturbed flow on vascular endothelium: pathophysiological basis and clinical perspectives. Physiol Rev. 2011;91:327–87. 21248169 10.1152/physrev.00047.2009 PMC3844671

[55] Okuda K, Khan MY, Skurnick J, Kimura M, Aviv H, Aviv A. Telomere attrition of the human abdominal aorta: relationships with age and atherosclerosis. Atherosclerosis. 2000;152:391–8. 10998467 10.1016/s0021-9150(99)00482-7

[56] Kotla S, Vu HT, Ko KA, Endothelial senescence is induced by phosphorylation and nuclear export of telomeric repeat binding factor 2-interacting protein. JCI Insight. 2019;4:124867. 31045573 10.1172/jci.insight.124867 PMC6538340

[57] Warboys CM, de Luca A, Amini N, Disturbed flow promotes endothelial senescence via a p53-dependent pathway. Arterioscler Thromb Vasc Biol. 2014;34:985–95. 24651677 10.1161/ATVBAHA.114.303415

[58] VanderLaan PA, Reardon CA, Getz GS. Site specificity of atherosclerosis: site-selective responses to atherosclerotic modulators. Arterioscler Thromb Vasc Biol. 2004;24:12–22. 14604830 10.1161/01.ATV.0000105054.43931.f0

[59] Li Y, Liu Z, Han X, Dynamics of endothelial cell generation and turnover in arteries during homeostasis and diseases. Circulation. 2024;149:135–54. 38084582 10.1161/CIRCULATIONAHA.123.064301

[60] Jones RC, Karkanias J, Krasnow MA, The tabula sapiens: a multiple-organ, single-cell transcriptomic atlas of humans. Science. 2022;376:eabl4896. 35549404 10.1126/science.abl4896 PMC9812260

[61] Jacobsen K, Lund MB, Shim J, Diverse cellular architecture of atherosclerotic plaque derives from clonal expansion of a few medial SMCs. JCI Insight. 2017;2:95890. 28978793 10.1172/jci.insight.95890 PMC5841865

[62] Misra A, Feng Z, Chandran RR, Integrin beta3 regulates clonality and fate of smooth muscle-derived atherosclerotic plaque cells. Nat Commun. 2018;9:2073. 29802249 10.1038/s41467-018-04447-7 PMC5970166

[63] Chappell J, Harman JL, Narasimhan VM, Extensive proliferation of a subset of differentiated, yet plastic, medial vascular smooth muscle cells contributes to neointimal formation in mouse injury and atherosclerosis models. Circ Res. 2016;119:1313–23. 27682618 10.1161/CIRCRESAHA.116.309799 PMC5149073

[64] McDonald AI, Shirali AS, Aragón R, Endothelial regeneration of large vessels is a biphasic process driven by local cells with distinct proliferative capacities. Cell Stem Cell. 2018;23:210–25.e6. 30075129 10.1016/j.stem.2018.07.011 PMC6178982

[65] Mahoney SA, Venkatasubramanian R, Darrah MA, Intermittent supplementation with fisetin improves arterial function in old mice by decreasing cellular senescence. Aging Cell. 2024;23:e14060. 38062873 10.1111/acel.14060 PMC10928570

[66] Conboy MJ, Conboy IM, Rando TA. Heterochronic parabiosis: historical perspective and methodological considerations for studies of aging and longevity. Aging Cell. 2013;12:525–30. 23489470 10.1111/acel.12065 PMC4072458

[67] Jeon OH, Mehdipour M, Gil TH, Systemic induction of senescence in young mice after single heterochronic blood exchange. Nat Metab. 2022;4:995–1006. 35902645 10.1038/s42255-022-00609-6 PMC9945470

[68] Craighead DH, Heinbockel TC, Freeberg KA, Time-efficient inspiratory muscle strength training lowers blood pressure and improves endothelial function, NO bioavailability, and oxidative stress in midlife/older adults with above-normal blood pressure. J Am Heart Assoc. 2021;10:e020980. 34184544 10.1161/JAHA.121.020980 PMC8403283

[69] Rossman MJ, Gioscia-Ryan RA, Santos-Parker JR, Inorganic nitrite supplementation improves endothelial function with aging: translational evidence for suppression of mitochondria-derived oxidative stress. Hypertension. 2021;77:1212–22. 33641356 10.1161/HYPERTENSIONAHA.120.16175 PMC7946806

[70] Mahoney SA, VanDongen NS, Greenberg NT, Role of the circulating milieu in age-related arterial dysfunction: a novel ex vivo approach. Am J Physiol Heart Circ Physiol. 2024;326:H1279–90. 38517225 10.1152/ajpheart.00014.2024 PMC11380963

[71] Kiss T, Tarantini S, Csipo T, Circulating anti-geronic factors from heterochonic parabionts promote vascular rejuvenation in aged mice: transcriptional footprint of mitochondrial protection, attenuation of oxidative stress, and rescue of endothelial function by young blood. Geroscience. 2020;42:727–48. 32172434 10.1007/s11357-020-00180-6 PMC7205954

[72] Bloom SI, Islam MT, Lesniewski LA, Donato AJ. Mechanisms and consequences of endothelial cell senescence. Nat Rev Cardiol. 2023;20:38–51. 35853997 10.1038/s41569-022-00739-0 PMC10026597

[73] Lee AH, Ghosh D, Koh IL, Dawson MR. Senescence-associated exosomes transfer miRNA-induced fibrosis to neighboring cells. Aging. 2023;15:1237–56. 36842089 10.18632/aging.204539 PMC10042705

[74] Terlecki-Zaniewicz L, Lämmermann I, Latreille J, Small extracellular vesicles and their miRNA cargo are anti-apoptotic members of the senescence-associated secretory phenotype. Aging. 2018;10:1103–32. 29779019 10.18632/aging.101452 PMC5990398

[75] Amersfoort J, Eelen G, Carmeliet P. Immunomodulation by endothelial cells - partnering up with the immune system? Nat Rev Immunol. 2022;22:576–88. 35288707 10.1038/s41577-022-00694-4 PMC8920067

[76] Uryga AK, Grootaert MOJ, Garrido AM, Telomere damage promotes vascular smooth muscle cell senescence and immune cell recruitment after vessel injury. Commun Biol. 2021;4:611. 34021256 10.1038/s42003-021-02123-z PMC8140103

[77] Mazan-Mamczarz K, Tsitsipatis D, Carr A, Single-cell and spatial transcriptomics uncovers the role of senescent vascular cells in pathological arterial remodeling during atherosclerosis. Research Square Platform LLC; 2023. 10.1038/s43587-025-00889-z PMC12323397 40660002

[78] Krouwer VJ, Hekking LH, Langelaar-Makkinje M, Regan-Klapisz E, Post JA. Endothelial cell senescence is associated with disrupted cell-cell junctions and increased monolayer permeability. Vasc Cell. 2012;4:12. 22929066 10.1186/2045-824X-4-12 PMC3527188

[79] Mun GI, Boo YC. Identification of CD44 as a senescence-induced cell adhesion gene responsible for the enhanced monocyte recruitment to senescent endothelial cells. Am J Physiol Heart Circ Physiol. 2010;298:H2102–11. 20382854 10.1152/ajpheart.00835.2009

[80] Real MGC, Falcione SR, Boghozian R, Endothelial cell senescence effect on the blood-brain barrier in stroke and cognitive impairment. Neurology. 2024;103:e210063. 39541552 10.1212/WNL.0000000000210063

[81] Beidokhti M, Villalba N, Ma Y, Reynolds A, Villamil JH, Yuan SY. Lung endothelial cell senescence impairs barrier function and promotes neutrophil adhesion and migration. Geroscience. 2025. Online ahead of print 10.1007/s11357-025-01517-9 PMC12181458 39815038

[82] Tang L, Zhang C, Yang Q, Melatonin maintains inner blood-retinal barrier via inhibition of p38/TXNIP/NF-κB pathway in diabetic retinopathy. J Cell Physiol. 2021;236:5848–64. 33432588 10.1002/jcp.30269

[83] Parrinello S, Samper E, Krtolica A, Goldstein J, Melov S, Campisi J. Oxygen sensitivity severely limits the replicative lifespan of murine fibroblasts. Nat Cell Biol. 2003;5:741–7. 12855956 10.1038/ncb1024 PMC4940195

[84] Busuttil RA, Rubio M, Dollé ME, Campisi J, Vijg J. Oxygen accelerates the accumulation of mutations during the senescence and immortalization of murine cells in culture. Aging Cell. 2003;2:287–94. 14677631 10.1046/j.1474-9728.2003.00066.x

[85] Roos CM, Zhang B, Palmer AK, Chronic senolytic treatment alleviates established vasomotor dysfunction in aged or atherosclerotic mice. Aging Cell. 2016;15:973–7. 26864908 10.1111/acel.12458 PMC5013022

[86] Ko CH, Takahashi JS. Molecular components of the mammalian circadian clock. Hum Mol Genet. 2006;15:R271–7. 16987893 10.1093/hmg/ddl207

[87] Bunger MK, Wilsbacher LD, Moran SM, Mop3 is an essential component of the master circadian pacemaker in mammals. Cell. 2000;103:1009–17. 11163178 10.1016/s0092-8674(00)00205-1 PMC3779439

[88] Rey G, Cesbron F, Rougemont J, Reinke H, Brunner M, Naef F. Genome-wide and phase-specific DNA-binding rhythms of BMAL1 control circadian output functions in mouse liver. PLoS Biol. 2011;9:e1000595. 21364973 10.1371/journal.pbio.1000595 PMC3043000

[89] Menet JS, Pescatore S, Rosbash M. CLOCK:BMAL1 is a pioneer-like transcription factor. Genes Dev. 2014;28:8–13. 24395244 10.1101/gad.228536.113 PMC3894415

[90] Crnko S, Cour M, Van Laake LW, Lecour S. Vasculature on the clock: Circadian rhythm and vascular dysfunction. Vascul Pharmacol. 2018;108:1–7. 29778521 10.1016/j.vph.2018.05.003

[91] Fan R, Peng X, Xie L, Importance of bmal1 in Alzheimer’s disease and associated aging-related diseases: mechanisms and interventions. Aging Cell. 2022;21:e13704. 36056774 10.1111/acel.13704 PMC9577946

[92] Bhatwadekar AD, Beli E, Diao Y, Conditional deletion of bmal1 accentuates microvascular and macrovascular injury. Am J Pathol. 2017;187:1426–35. 28432873 10.1016/j.ajpath.2017.02.014 PMC5455061

[93] Jachim SK, Zhong J, Ordog T, BMAL1 modulates senescence programming via AP-1. Aging. 2023;15:9984–10009. 37819791 10.18632/aging.205112 PMC10599731

[94] Rafikov R, Sun X, Rafikova O, Complex I dysfunction underlies the glycolytic switch in pulmonary hypertensive smooth muscle cells. Redox Biol. 2015;6:278–86. 26298201 10.1016/j.redox.2015.07.016 PMC4556771

[95] Wiley CD, Campisi J. From ancient pathways to aging cells-connecting metabolism and cellular senescence. Cell Metab. 2016;23:1013–21. 27304503 10.1016/j.cmet.2016.05.010 PMC4911819

[96] López-Otín C, Blasco MA, Partridge L, Serrano M, Kroemer G. Hallmarks of aging: an expanding universe. Cell. 2023;186:243–78. 36599349 10.1016/j.cell.2022.11.001

[97] der Bruggen MM, Spronck B, Delhaas T, Reesink KD, Schalkwijk CG. The putative role of methylglyoxal in arterial stiffening: a review. Heart Lung Circ. 2021;30:1681–93. 34393049 10.1016/j.hlc.2021.06.527

[98] Aragonès G, Rowan S, Francisco SG, The glyoxalase system in age-related diseases: nutritional intervention as anti-ageing strategy. Cells. 2021;10:1852. 34440621 10.3390/cells10081852 PMC8393707

[99] Halkoum R, Salnot V, Capallere C, Glyoxal induces senescence in human keratinocytes through oxidative stress and activation of the protein kinase B/FOXO3a/p27^KIP1^ pathway. J Invest Dermatol. 2022;142:2068–78.e7. 34971698 10.1016/j.jid.2021.12.022

[100] Li H, Zheng L, Chen C, Liu X, Zhang W. Brain senescence caused by elevated levels of reactive metabolite methylglyoxal on D-galactose-induced aging mice. Front Neurosci. 2019;13:1004. 31619960 10.3389/fnins.2019.01004 PMC6760031

[101] Ikeda Y, Inagi R, Miyata T, Glyoxalase I retards renal senescence. Am J Pathol. 2011;179:2810–21. 22001178 10.1016/j.ajpath.2011.08.023 PMC3260855

[102] Jo-Watanabe A, Ohse T, Nishimatsu H, Glyoxalase I reduces glycative and oxidative stress and prevents age-related endothelial dysfunction through modulation of endothelial nitric oxide synthase phosphorylation. Aging Cell. 2014;13:519–28. 24612481 10.1111/acel.12204 PMC4326886

[103] Ziegler DV, Martin N, Bernard D. Cellular senescence links mitochondria-ER contacts and aging. Commun Biol. 2021;4:1323. 34819602 10.1038/s42003-021-02840-5 PMC8613202

[104] Wang J, Bai Y, Zhao X, oxLDL-mediated cellular senescence is associated with increased NADPH oxidase p47phox recruitment to caveolae. Biosci Rep. 2018;38:BSR20180283. 29695496 10.1042/BSR20180283 PMC5997791

[105] Yu S, Zhang L, Liu C, Yang J, Zhang J, Huang L. PACS2 is required for ox-LDL-induced endothelial cell apoptosis by regulating mitochondria-associated ER membrane formation and mitochondrial Ca^2+^ elevation. Exp Cell Res. 2019;379:191–202. 30970236 10.1016/j.yexcr.2019.04.002

[106] Ziegler DV, Vindrieux D, Goehrig D, Calcium channel ITPR2 and mitochondria-ER contacts promote cellular senescence and aging. Nat Commun. 2021;12:720. 33526781 10.1038/s41467-021-20993-z PMC7851384

[107] Bolinches-Amorós A, Mollá B, Pla-Martín D, Palau F, González-Cabo P. Mitochondrial dysfunction induced by frataxin deficiency is associated with cellular senescence and abnormal calcium metabolism. Front Cell Neurosci. 2014;8:124. 24860428 10.3389/fncel.2014.00124 PMC4026758

[108] Rodríguez LR, Calap-Quintana P, Lapeña-Luzón T, Oxidative stress modulates rearrangement of endoplasmic reticulum-mitochondria contacts and calcium dysregulation in a Friedreich’s ataxia model. Redox Biol. 2020;37:101762. 33128998 10.1016/j.redox.2020.101762 PMC7585950

[109] Piera-Velazquez S, Jimenez SA. Endothelial to mesenchymal transition: role in physiology and in the pathogenesis of human diseases. Physiol Rev. 2019;99:1281–324. 30864875 10.1152/physrev.00021.2018 PMC6734087

[110] Ramadhiani R, Ikeda K, Hirata KI, Emoto N. Endothelial cell senescence exacerbates pulmonary fibrosis potentially through accelerated endothelial to mesenchymal transition. Kobe J Med Sci. 2021;67:E84–91. 35367994 PMC9673883

[111] Zhu J, Angelov S, Alp Yildirim I, Loss of transforming growth factor beta signaling in aortic smooth muscle cells causes endothelial dysfunction and aortic hypercontractility. Arterioscler Thromb Vasc Biol. 2021;41:1956–71. 33853348 10.1161/ATVBAHA.121.315878 PMC8159907

[112] Fleenor BS, Marshall KD, Durrant JR, Lesniewski LA, Seals DR. Arterial stiffening with ageing is associated with transforming growth factor-β1-related changes in adventitial collagen: reversal by aerobic exercise. J Physiol. 2010;588:3971–82. 20807791 10.1113/jphysiol.2010.194753 PMC3000586

[113] Fleenor BS, Marshall KD, Rippe C, Seals DR. Replicative aging induces endothelial to mesenchymal transition in human aortic endothelial cells: potential role of inflammation. J Vasc Res. 2012;49:59–64. 21985896 10.1159/000329681 PMC3214888

[114] Hamczyk MR, Nevado RM, Barettino A, Fuster V, Andrés V. Biological versus chronological aging: JACC focus seminar. J Am Coll Cardiol. 2020;75:919–30. 32130928 10.1016/j.jacc.2019.11.062

[115] Sharma R, Oni OA, Gupta K, Normalization of testosterone level is associated with reduced incidence of myocardial infarction and mortality in men. Eur Heart J. 2015;36:2706–15. 26248567 10.1093/eurheartj/ehv346

[116] Vlachopoulos C, Ioakeimidis N, Miner M, Testosterone deficiency: a determinant of aortic stiffness in men. Atherosclerosis. 2014;233:278–83. 24529157 10.1016/j.atherosclerosis.2013.12.010

[117] Yildiz O, Seyrek M. Vasodilating mechanisms of testosterone. Exp Clin Endocrinol Diabetes. 2007;115:1–6. 17286226 10.1055/s-2007-949657

[118] Novella S, Dantas AP, Segarra G, Gathering of aging and estrogen withdrawal in vascular dysfunction of senescent accelerated mice. Exp Gerontol. 2010;45:868–74. 20708673 10.1016/j.exger.2010.07.007

[119] Maeda M, Hayashi T, Mizuno N, Hattori Y, Kuzuya M. Intermittent high glucose implements stress-induced senescence in human vascular endothelial cells: role of superoxide production by NADPH oxidase. PLoS One. 2015;10:e0123169. 25879533 10.1371/journal.pone.0123169 PMC4400006

[120] Farhat N, Thorin-Trescases N, Voghel G, Stress-induced senescence predominates in endothelial cells isolated from atherosclerotic chronic smokers. Can J Physiol Pharmacol. 2008;86:761–9. 19011671 10.1139/Y08-082 PMC3701584

[121] Carreras A, Zhang SX, Peris E, Chronic sleep fragmentation induces endothelial dysfunction and structural vascular changes in mice. Sleep. 2014;37:1817–24. 25364077 10.5665/sleep.4178 PMC4196065

[122] Satyjeet F, Naz S, Kumar V, Psychological stress as a risk factor for cardiovascular disease: a case-control study. Cureus. 2020;12:e10757. 33150108 10.7759/cureus.10757 PMC7603890

[123] Vancheri F, Longo G, Vancheri E, Henein MY. Mental stress and cardiovascular health-part I. J Clin Med. 2022;11:3353. 35743423 10.3390/jcm11123353 PMC9225328

[124] Sara JDS, Toya T, Ahmad A, Mental stress and its effects on vascular health. Mayo Clin Proc. 2022;97:951–90. 35512885 10.1016/j.mayocp.2022.02.004 PMC9058928

[125] Sara JDS, Lerman LO, Lerman A. What can biologic aging tell us about the effects of mental stress on vascular health. Hypertension. 2023;80:2515–22. 37814855 10.1161/HYPERTENSIONAHA.123.19418

[126] Hayashi T, Yano K, Matsui-Hirai H, Yokoo H, Hattori Y, Iguchi A. Nitric oxide and endothelial cellular senescence. Pharmacol Ther. 2008;120:333–9. 18930078 10.1016/j.pharmthera.2008.09.002

[127] Lin YF, Wang LY, Chen CS, Li CC, Hsiao YH. Cellular senescence as a driver of cognitive decline triggered by chronic unpredictable stress. Neurobiol Stress. 2021;15:100341. 34095365 10.1016/j.ynstr.2021.100341 PMC8163993

[128] Rentscher KE, Carroll JE, Repetti RL, Cole SW, Reynolds BM, Robles TF. Chronic stress exposure and daily stress appraisals relate to biological aging marker p16^INK4a^. Psychoneuroendocrinology. 2019;102:139–48. 30557761 10.1016/j.psyneuen.2018.12.006 PMC6420375

[129] Jimenez DE, Alegría M, Chen CN, Chan D, Laderman M. Prevalence of psychiatric illnesses in older ethnic minority adults. J Am Geriatr Soc. 2010;58:256–64. 20374401 10.1111/j.1532-5415.2009.02685.x PMC2854540

[130] Pietrzak RH, Goldstein RB, Southwick SM, Grant BF. Prevalence and axis I comorbidity of full and partial posttraumatic stress disorder in the United States: results from wave 2 of the national epidemiologic survey on alcohol and related conditions. J Anxiety Disord. 2011;25:456–65. 21168991 10.1016/j.janxdis.2010.11.010 PMC3051041

[131] Kaiser A, Cook JM, Glick DM, Moye J. Posttraumatic stress disorder in older adults: a conceptual review. Clin Gerontol. 2019;42:359–76. 30422749 10.1080/07317115.2018.1539801 PMC6666306

[132] Wolf EJ, Morrison FG. Traumatic stress and accelerated cellular aging: from epigenetics to cardiometabolic disease. Curr Psychiatry Rep. 2017;19:75. 28852965 10.1007/s11920-017-0823-5 PMC5588711

[133] Lohr JB, Palmer BW, Eidt CA, Is post-traumatic stress disorder associated with premature senescence? A review of the literature. Am J Geriatr Psychiatry. 2015;23:709–25. 25959921 10.1016/j.jagp.2015.04.001 PMC4568841

[134] Wang Y, Boerma M, Zhou D. Ionizing radiation-induced endothelial cell senescence and cardiovascular diseases. Radiat Res. 2016;186:153–61. 27387862 10.1667/RR14445.1 PMC4997805

[135] Zheng X, Liu Z, Bin Y, Ionizing radiation induces vascular smooth muscle cell senescence through activating NF-κB/CTCF/p16 pathway. Biochim Biophys Acta Mol Basis Dis. 2024;1870:166994. 38141838 10.1016/j.bbadis.2023.166994

[136] Yamamoto Y, Minami M, Yoshida K, Irradiation accelerates plaque formation and cellular senescence in flow-altered carotid arteries of apolipoprotein E knock-out mice. J Am Heart Assoc. 2021;10:e020712. 34227406 10.1161/JAHA.120.020712 PMC8483483

[137] Fernández-Alvarez V, Nieto CS, Alvarez FL. Arterial stiffness as an ultrasound biomarker of radiation-induced carotid artery disease. Vasa. 2021;50:348–55. 34102858 10.1024/0301-1526/a000956

[138] Bansal N, Amdani SM, Hutchins KK, Lipshultz SE. Cardiovascular disease in survivors of childhood cancer. Curr Opin Pediatr. 2018;30:628–38. 30124579 10.1097/MOP.0000000000000675

[139] Lipshultz SE, Karnik R, Sambatakos P, Franco VI, Ross SW, Miller TL. Anthracycline-related cardiotoxicity in childhood cancer survivors. Curr Opin Cardiol. 2014;29:103–12. 24284979 10.1097/HCO.0000000000000034

[140] Cappetta D, Rossi F, Piegari E, Doxorubicin targets multiple players: a new view of an old problem. Pharmacol Res. 2018;127:4–14. 28336372 10.1016/j.phrs.2017.03.016

[141] Bourlon MT, Velazquez HE, Hinojosa J, Immunosenescence profile and expression of the aging biomarker (p16^INK4a^) in testicular cancer survivors treated with chemotherapy. BMC Cancer. 2020;20:882. 32928147 10.1186/s12885-020-07383-2 PMC7491179

[142] Smitherman AB, Wood WA, Mitin N, Accelerated aging among childhood, adolescent, and young adult cancer survivors is evidenced by increased expression of p16^INK4a^ and frailty. Cancer. 2020;126:4975–83. 32830315 10.1002/cncr.33112 PMC7607511

[143] Muss HB, Smitherman A, Wood WA, p16 a biomarker of aging and tolerance for cancer therapy. Transl Cancer Res. 2020;9:5732–42. 35117935 10.21037/tcr.2020.03.39 PMC8797727

[144] Clayton ZS, Hutton DA, Mahoney SA, Seals DR. Anthracycline chemotherapy-mediated vascular dysfunction as a model of accelerated vascular aging. Aging Cancer. 2021;2:45–69. 34212156 10.1002/aac2.12033 PMC8240486

[145] Hutton D, Brunt V, Mahoney S, Cellular senescence mediates doxorubicin-induced arterial dysfunction via activation of mitochondrial oxidative stress and the mammalian target of rapamycin. FASEB J. 2021;35:00283.

[146] Jain D, Ahmad T, Cairo M, Aronow W. Cardiotoxicity of cancer chemotherapy: identification, prevention and treatment. Ann Transl Med. 2017;5:348. 28936442 10.21037/atm.2017.06.35 PMC5599271

[147] Axel DI, Kunert W, Göggelmann C, Paclitaxel inhibits arterial smooth muscle cell proliferation and migration in vitro and in vivo using local drug delivery. Circulation. 1997;96:636–45. 9244237 10.1161/01.cir.96.2.636

[148] Ahire C, Nyul-Toth A, DelFavero J, Accelerated cerebromicrovascular senescence contributes to cognitive decline in a mouse model of paclitaxel (Taxol)-induced chemobrain. Aging Cell. 2023;22:e13832. 37243381 10.1111/acel.13832 PMC10352561

[149] Kanmogne GD. HIV Infection, antiretroviral drugs, and the vascular endothelium. Cells. 2024;13:672. 38667287 10.3390/cells13080672 PMC11048826

[150] Gianesin K, Noguera-Julian A, Zanchetta M, Premature aging and immune senescence in HIV-infected children. AIDS. 2016;30:1363–73. 26990630 10.1097/QAD.0000000000001093 PMC4867984

[151] Kuehnemann C, Hughes JB, Desprez PY, Melov S, Wiley CD, Campisi J. Antiretroviral protease inhibitors induce features of cellular senescence that are reversible upon drug removal. Aging Cell. 2023;22:e13750. 36539941 10.1111/acel.13750 PMC9835573

[152] Cohen J, D’Agostino L, Tuzer F, Torres C. HIV antiretroviral therapy drugs induce premature senescence and altered physiology in HUVECs. Mech Ageing Dev. 2018;175:74–82. 30055190 10.1016/j.mad.2018.07.008 PMC6133242

[153] Kaur G, Sohanur Rahman M, Shaikh S, Emerging roles of senolytics/senomorphics in HIV-related co-morbidities. Biochem Pharmacol. 2024;228:116179. 38556028 10.1016/j.bcp.2024.116179 PMC11410549

[154] Matthews C, Gorenne I, Scott S, Vascular smooth muscle cells undergo telomere-based senescence in human atherosclerosis: effects of telomerase and oxidative stress. Circ Res. 2006;99:156–64. 16794190 10.1161/01.RES.0000233315.38086.bc

[155] Wang J, Uryga AK, Reinhold J, Vascular smooth muscle cell senescence promotes atherosclerosis and features of plaque vulnerability. Circulation. 2015;132:1909–19. 26416809 10.1161/CIRCULATIONAHA.115.016457

[156] van der Feen DE, Bossers GPL, Hagdorn QAJ, Cellular senescence impairs the reversibility of pulmonary arterial hypertension. Sci Transl Med. 2020;12:eaaw4974. 32727916 10.1126/scitranslmed.aaw4974 PMC7891555

[157] Chen HZ, Wang F, Gao P, Age-associated sirtuin 1 reduction in vascular smooth muscle links vascular senescence and inflammation to abdominal aortic aneurysm. Circ Res. 2016;119:1076–88. 27650558 10.1161/CIRCRESAHA.116.308895 PMC6546422

[158] Tyrrell DJ, Chen J, Li BY, Aging alters the aortic proteome in health and thoracic aortic aneurysm. Arterioscler Thromb Vasc Biol. 2022;42:1060–76. 35510553 10.1161/ATVBAHA.122.317643 PMC9339483

[159] Minamino T, Komuro I. Vascular cell senescence: contribution to atherosclerosis. Circ Res. 2007;100:15–26. 17204661 10.1161/01.RES.0000256837.40544.4a

[160] Sun Y, Wang X, Liu T, Zhu X, Pan X. The multifaceted role of the SASP in atherosclerosis: from mechanisms to therapeutic opportunities. Cell Biosci. 2022;12:74. 35642067 10.1186/s13578-022-00815-5 PMC9153125

[161] Lu H, Du W, Ren L, Vascular smooth muscle cells in aortic aneurysm: from genetics to mechanisms. J Am Heart Assoc. 2021;10:e023601. 34796717 10.1161/JAHA.121.023601 PMC9075263

[162] Noureddine H, Gary-Bobo G, Alifano M, Pulmonary artery smooth muscle cell senescence is a pathogenic mechanism for pulmonary hypertension in chronic lung disease. Circ Res. 2011;109:543–53. 21719760 10.1161/CIRCRESAHA.111.241299 PMC3375237

[163] Gonzalo S, Kreienkamp R, Askjaer P. Hutchinson-gilford progeria syndrome: a premature aging disease caused by LMNA gene mutations. Ageing Res Rev. 2017;33:18–29. 27374873 10.1016/j.arr.2016.06.007 PMC5195863

[164] Xu Q, Mojiri A, Boulahouache L, Morales E, Walther BK, Cooke JP. Vascular senescence in progeria: role of endothelial dysfunction. Eur Heart J Open. 2022;2:oeac047. 36117952 10.1093/ehjopen/oeac047 PMC9472787

[165] Wong A, Kieu T, Robbins PD. The Ercc1-/Δ mouse model of accelerated senescence and aging for identification and testing of novel senotherapeutic interventions. Aging. 2020;12:24481–3. 33353886 10.18632/aging.202321 PMC7803498

[166] Ataei Ataabadi E, Golshiri K, van der Linden J, Vascular ageing features caused by selective DNA damage in smooth muscle cell. Oxid Med Cell Longev. 2021;2021:2308317. 34504640 10.1155/2021/2308317 PMC8423575

[167] Shamanna RA, Croteau DL, Lee JH, Bohr VA. Recent advances in understanding werner syndrome. F1000Res. 2017;6:1779. 29043077 10.12688/f1000research.12110.1 PMC5621106

[168] Kato H, Maezawa Y. Atherosclerosis and cardiovascular diseases in progeroid syndromes. J Atheroscler Thromb. 2022;29:439–47. 34511576 10.5551/jat.RV17061 PMC9100459

[169] Norwood TH, Hoehn H, Salk D, Martin GM. Cellular aging in Werner’s syndrome: a unique phenotype? J Invest Dermatol. 1979;73:92–6. 448183 10.1111/1523-1747.ep12532778

[170] Paul SK, Oshima M, Patil A, Retrotransposons in Werner syndrome-derived macrophages trigger type I interferon-dependent inflammation in an atherosclerosis model. Nat Commun. 2024;15:4772. 38858384 10.1038/s41467-024-48663-w PMC11164933

[171] Zhu JL, Hasle H, Correa A, Survival among people with down syndrome: a nationwide population-based study in denmark. Genet Med. 2013;15:64–9. 22878506 10.1038/gim.2012.93 PMC4532298

[172] Cappelli-Bigazzi M, Santoro G, Battaglia C, Endothelial cell function in patients with down’s syndrome. Am J Cardiol. 2004;94:392–5. 15276117 10.1016/j.amjcard.2004.04.047

[173] Koike MA, Green KN, Blurton-Jones M, Laferla FM. Oligemic hypoperfusion differentially affects tau and amyloid-β. Am J Pathol. 2010;177:300–10. 20472896 10.2353/ajpath.2010.090750 PMC2893673

[174] Garcia-Alloza M, Gregory J, Kuchibhotla KV, Cerebrovascular lesions induce transient β-amyloid deposition. Brain. 2011;134:3697–707. 22120142 10.1093/brain/awr300 PMC3235567

[175] Wee SO, Rosenberg AJ, Kanokwan B, Griffith G, Baynard T, Fernhall B. Carotid vascular blood flow in individuals with down syndrome following low body negative pressure challenge. FASEB J. 2017;31:840.21.27856557

[176] Meharena HS, Marco A, Dileep V, Down-syndrome-induced senescence disrupts the nuclear architecture of neural progenitors. Cell Stem Cell. 2022;29:116–30.e7. 34995493 10.1016/j.stem.2021.12.002 PMC8805993

[177] Gimeno A, García-Giménez JL, Audí L, Decreased cell proliferation and higher oxidative stress in fibroblasts from Down Syndrome fetuses. Preliminary study. Biochim Biophys Acta. 2014;1842:116–25. 24184606 10.1016/j.bbadis.2013.10.014

[178] de Arruda Cardoso Smith M, Borsatto-Galera B, Feller RI, Telomeres on chromosome 21 and aging in lymphocytes and gingival fibroblasts from individuals with Down syndrome. J Oral Sci. 2004;46:171–7. 15508750 10.2334/josnusd.46.171

[179] Zhang L, Pitcher LE, Prahalad V, Niedernhofer LJ, Robbins PD. Targeting cellular senescence with senotherapeutics: senolytics and senomorphics. FEBS J. 2023;290:1362–83. 35015337 10.1111/febs.16350

[180] Chen XK, Yi ZN, Wong GTC, Is exercise a senolytic medicine? A systematic review. Aging Cell. 2021;20:e13294. 33378138 10.1111/acel.13294 PMC7811843

[181] Lynch DH, Petersen CL, Stewart D, Changes in senescence markers after a weight loss intervention in older adults with obesity Arch Gerontol Geriatr 2025. p. 105685. 39541752 10.1016/j.archger.2024.105685 PMC11616393

[182] Wang B, Han J, Elisseeff JH, Demaria M. The senescence-associated secretory phenotype and its physiological and pathological implications. Nat Rev Mol Cell Biol. 2024;25:958–78. 38654098 10.1038/s41580-024-00727-x

[183] Kirkland JL, Tchkonia T. Senolytic drugs: from discovery to translation. J Intern Med. 2020;288:518–36. 32686219 10.1111/joim.13141 PMC7405395

[184] Zhu Y, Tchkonia T, Fuhrmann-Stroissnigg H, Identification of a novel senolytic agent, navitoclax, targeting the Bcl-2 family of anti-apoptotic factors. Aging Cell. 2016;15:428–35. 26711051 10.1111/acel.12445 PMC4854923

[185] Wilson WH, O’Connor OA, Czuczman MS, Navitoclax, a targeted high-affinity inhibitor of BCL-2, in lymphoid malignancies: a phase 1 dose-escalation study of safety, pharmacokinetics, pharmacodynamics, and antitumour activity. Lancet Oncol. 2010;11:1149–59. 21094089 10.1016/S1470-2045(10)70261-8 PMC3025495

[186] Karnewar S, Karnewar V, Shankman LS, Owens GK. Treatment of advanced atherosclerotic mice with ABT-263 reduced indices of plaque stability and increased mortality. JCI Insight. 2024;9:e173863. 38258907 10.1172/jci.insight.173863 PMC10906456

[187] Garrido AM, Kaistha A, Uryga AK, Efficacy and limitations of senolysis in atherosclerosis. Cardiovasc Res. 2022;118:1713–27. 34142149 10.1093/cvr/cvab208 PMC9215197

[188] Childs BG, Zhang C, Shuja F, Senescent cells suppress innate smooth muscle cell repair functions in atherosclerosis. Nat Aging. 2021;1:698–714. 34746803 10.1038/s43587-021-00089-5 PMC8570576

[189] Justice JN, Nambiar AM, Tchkonia T, Senolytics in idiopathic pulmonary fibrosis: results from a first-in-human, open-label, pilot study. EBioMedicine. 2019;40:554–63. 30616998 10.1016/j.ebiom.2018.12.052 PMC6412088

[190] Gonzales MM, Garbarino VR, Kautz T, Senolytic therapy to modulate the progression of Alzheimer’s disease (SToMP-AD) - outcomes from the first clinical trial of senolytic therapy for Alzheimer’s disease. Res Sq. 2023;rs.3.rs-2809973.

[191] Parvizi M, Franchi F, Arendt BK, Ebtehaj S, Rodriguez-Porcel M, Lanza IR. Senolytic agents lessen the severity of abdominal aortic aneurysm in aged mice. Exp Gerontol. 2021;151:111416. 34022272 10.1016/j.exger.2021.111416 PMC11443445

[192] Yousefzadeh MJ, Zhu Y, McGowan SJ, Fisetin is a senotherapeutic that extends health and lifespan. EBioMedicine. 2018;36:18–28. 30279143 10.1016/j.ebiom.2018.09.015 PMC6197652

[193] Mahoney S, Ciotlos S, Darrah M, 25-Hydroxycholesterol reduces aortic cellular senescence and stiffness in old mice. Physiology. 2023;38:5711505.

[194] Johmura Y, Yamanaka T, Omori S, Senolysis by glutaminolysis inhibition ameliorates various age-associated disorders. Science. 2021;371:265–70. 33446552 10.1126/science.abb5916

[195] Suda M, Shimizu I, Katsuumi G, Senolytic vaccination improves normal and pathological age-related phenotypes and increases lifespan in progeroid mice. Nat Aging. 2021;1:1117–26. 37117524 10.1038/s43587-021-00151-2

[196] Ota H, Eto M, Kano MR, Induction of endothelial nitric oxide synthase, SIRT1, and catalase by statins inhibits endothelial senescence through the Akt pathway. Arterioscler Thromb Vasc Biol. 2010;30:2205–11. 20705918 10.1161/ATVBAHA.110.210500

[197] Abdelgawad IY, Agostinucci K, Sadaf B, Grant MKO, Zordoky BN. Metformin mitigates SASP secretion and LPS-triggered hyper-inflammation in Doxorubicin-induced senescent endothelial cells. Front Aging. 2023;4:1170434. 37168843 10.3389/fragi.2023.1170434 PMC10164964

[198] Oeseburg H, de Boer RA, Buikema H, van der Harst P, van Gilst WH, Silljé HH. Glucagon-like peptide 1 prevents reactive oxygen species-induced endothelial cell senescence through the activation of protein kinase A. Arterioscler Thromb Vasc Biol. 2010;30:1407–14. 20448207 10.1161/ATVBAHA.110.206425

[199] Zhao L, Li AQ, Zhou TF, Zhang MQ, Qin XM. Exendin-4 alleviates angiotensin II-induced senescence in vascular smooth muscle cells by inhibiting Rac1 activation via a cAMP/PKA-dependent pathway. Am J Physiol Cell Physiol. 2014;307:C1130–41. 25298426 10.1152/ajpcell.00151.2014

[200] Bode-Böger SM, Martens-Lobenhoffer J, Täger M, Schröder H, Scalera F. Aspirin reduces endothelial cell senescence. Biochem Biophys Res Commun. 2005;334:1226–32. 16039999 10.1016/j.bbrc.2005.07.014

[201] Xia L, Wang XX, Hu XS, Resveratrol reduces endothelial progenitor cells senescence through augmentation of telomerase activity by Akt-dependent mechanisms. Br J Pharmacol. 2008;155:387–94. 18587418 10.1038/bjp.2008.272 PMC2567879

[202] Csiszar A, Sosnowska D, Wang M, Lakatta EG, Sonntag WE, Ungvari Z. Age-associated proinflammatory secretory phenotype in vascular smooth muscle cells from the non-human primate Macaca mulatta: reversal by resveratrol treatment. J Gerontol A Biol Sci Med Sci. 2012;67:811–20. 22219513 10.1093/gerona/glr228 PMC3536544

[203] Lesniewski LA, Seals DR, Walker AE, Dietary rapamycin supplementation reverses age-related vascular dysfunction and oxidative stress, while modulating nutrient-sensing, cell cycle, and senescence pathways. Aging Cell. 2017;16:17–26. 27660040 10.1111/acel.12524 PMC5242306

[204] Sasaki N, Itakura Y, Toyoda M. Rapamycin promotes endothelial-mesenchymal transition during stress-induced premature senescence through the activation of autophagy. Cell Commun Signal. 2020;18:43. 32164764 10.1186/s12964-020-00533-w PMC7069020

[205] Schoenwaelder SM, Jarman KE, Gardiner EE, Bcl-x_L_-inhibitory BH3 mimetics can induce a transient thrombocytopathy that undermines the hemostatic function of platelets. Blood. 2011;118:1663–74. 21673344 10.1182/blood-2011-04-347849

[206] Breccia M, Molica M, Alimena G. How tyrosine kinase inhibitors impair metabolism and endocrine system function: a systematic updated review. Leuk Res. 2014;38:1392–8. 25449685 10.1016/j.leukres.2014.09.016

[207] Crespo-Garcia S, Tsuruda PR, Dejda A, Pathological angiogenesis in retinopathy engages cellular senescence and is amenable to therapeutic elimination via BCL-xL inhibition. Cell Metab. 2021;33:818–32.e7. 33548171 10.1016/j.cmet.2021.01.011

[208] Crespo-Garcia S, Fournier F, Diaz-Marin R, Therapeutic targeting of cellular senescence in diabetic macular edema: preclinical and phase 1 trial results. Nat Med. 2024;30:443–54. 38321220 10.1038/s41591-024-02802-4

[209] Moiseeva V, Cisneros A, Sica V, Senescence atlas reveals an aged-like inflamed niche that blunts muscle regeneration. Nature. 2023;613:169–78. 36544018 10.1038/s41586-022-05535-x PMC9812788

[210] Wang B, Wang L, Gasek NS, An inducible p21-Cre mouse model to monitor and manipulate p21-highly-expressing senescent cells in vivo. Nat Aging. 2021;1:962–73. 35024619 10.1038/s43587-021-00107-6 PMC8746571

[211] Li M, Wang D, Liu Z, Assessing the effects of aging on the renal endothelial cell landscape using single-cell RNA sequencing. Front Genet. 2023;14:1175716. 37214419 10.3389/fgene.2023.1175716 PMC10196692

[212] Cohen C, Le Goff O, Soysouvanh F, Glomerular endothelial cell senescence drives age-related kidney disease through PAI-1. EMBO Mol Med. 2021;13:e14146. 34725920 10.15252/emmm.202114146 PMC8573606

[213] Amor C, Fernández-Maestre I, Chowdhury S, Prophylactic and long-lasting efficacy of senolytic CAR T cells against age-related metabolic dysfunction. Nat Aging. 2024;4:336–49. 38267706 10.1038/s43587-023-00560-5 PMC10950785

[214] Grosse L, Wagner N, Emelyanov A, Defined p16^High^ senescent cell types are indispensable for mouse healthspan. Cell Metab. 2020;32:87–99.e6. 32485135 10.1016/j.cmet.2020.05.002

[215] Omori S, Wang TW, Johmura Y, Generation of a p16 reporter mouse and its use to characterize and target p16^High^ cells in vivo. Cell Metab. 2020;32:814–28.e6. 32949498 10.1016/j.cmet.2020.09.006

[216] Ozawa M, Mori H, Endo T, Age-related decline in spermatogenic activity accompanied with endothelial cell senescence in male mice. iScience. 2023;26:108456. 38077127 10.1016/j.isci.2023.108456 PMC10700819

[217] Kiss T, Nyúl-Tóth Á, Balasubramanian P, Single-cell RNA sequencing identifies senescent cerebromicrovascular endothelial cells in the aged mouse brain. Geroscience. 2020;42:429–44. 32236824 10.1007/s11357-020-00177-1 PMC7205992

[218] Ximerakis M, Lipnick SL, Innes BT, Single-cell transcriptomic profiling of the aging mouse brain. Nat Neurosci. 2019;22:1696–708. 31551601 10.1038/s41593-019-0491-3

[219] Hussong SA, Banh AQ, Van Skike CE, Soluble pathogenic tau enters brain vascular endothelial cells and drives cellular senescence and brain microvascular dysfunction in a mouse model of tauopathy. Nat Commun. 2023;14:2367. 37185259 10.1038/s41467-023-37840-y PMC10126555

[220] Gulej R, Nyúl-Tóth Á, Ahire C, Elimination of senescent cells by treatment with Navitoclax/ABT263 reverses whole brain irradiation-induced blood-brain barrier disruption in the mouse brain. Geroscience. 2023;45:2983–3002. 37642933 10.1007/s11357-023-00870-x PMC10643778

[221] Wang L, Wang B, Gasek NS, Targeting *p21*^Cip1^ highly expressing cells in adipose tissue alleviates insulin resistance in obesity. Cell Metab. 2022;34:75–89.e8. 34813734 10.1016/j.cmet.2021.11.002 PMC8732323

[222] Lipskaia L, Breau M, Cayrou C, mTert induction in p21-positive cells counteracts capillary rarefaction and pulmonary emphysema. EMBO Rep. 2024;25:1650–84. 38424230 10.1038/s44319-023-00041-1 PMC10933469

[223] Culley MK, Zhao J, Tai YY, Frataxin deficiency promotes endothelial senescence in pulmonary hypertension. J Clin Invest. 2021;131:136459. 33905372 10.1172/JCI136459 PMC8159699

[224] Gao Z, Santos RB, Rupert J, Endothelial-specific telomerase inactivation causes telomere-independent cell senescence and multi-organ dysfunction characteristic of aging. Aging Cell. 2024;23:e14138. 38475941 10.1111/acel.14138 PMC11296101

[225] Barinda AJ, Ikeda K, Nugroho DB, Endothelial progeria induces adipose tissue senescence and impairs insulin sensitivity through senescence associated secretory phenotype. Nat Commun. 2020;11:481. 31980643 10.1038/s41467-020-14387-w PMC6981212

[226] Islam MT, Hall SA, Dutson T, Endothelial cell-specific reduction in mTOR ameliorates age-related arterial and metabolic dysfunction. Aging Cell. 2024;23:e14040. 38017701 10.1111/acel.14040 PMC10861194

